# DMAP-assisted sulfonylation as an efficient step for the methylation of primary amine motifs on solid support

**DOI:** 10.3762/bjoc.13.81

**Published:** 2017-05-03

**Authors:** Johnny N Naoum, Koushik Chandra, Dorit Shemesh, R Benny Gerber, Chaim Gilon, Mattan Hurevich

**Affiliations:** 1 Institute of Chemistry, The Hebrew University of Jerusalem, Edmond Safra Campus, Givat Ram, Jerusalem, 91904, Israel; 2 Fritz Haber Research Center, The Hebrew University of Jerusalem, Jerusalem 91904, Israel

**Keywords:** *N*-methylation, nucleophilic addition, solid phase, somatostatin, sulfonylation

## Abstract

Several multistep strategies were developed to ensure single methylation of amines on solid support. These strategies rely on the introduction of the *o*-NBS protecting/activating group as a key step. We found that the state-of-the-art strategies fail for the methylation of several primary amine motifs, largely due to inefficient sulfonylation. Here we show that using the superior nucleophilic base DMAP instead of the commonly used base collidine as a sulfonylation additive is essential for the introduction of the *o*-NBS group to these amine motifs. DFT calculations provide an explanation by showing that the energy barrier of the DMAP intermediate is significantly lower than the one of the collidine. We demonstrate that using DMAP as a sole additive in the sulfonylation step results in an overall effective and regioselective *N*-methylation. The method presented herein proved highly efficient in solid-phase synthesis of a somatostatin analogue bearing three *N*^α^-methylation sites that could not be synthesized using the previously described state-of-the-art methods.

## Introduction

Methylated amines and amides are common motifs found in natural and synthetic compounds, e.g., small molecules, peptides, and oligonucleotides [1–8]. Methylation of amines and amides has found many applications in the fields of pharmacological research, materials science, and in synthetic organic chemistry [9–16]. There is a major interest in developing synthetic methods for the preparation of methylated amines and amides [3]. The main challenge in the synthesis of *N*-methylated amines is to selectively prepare the mono-methylated product and to avoid the formation of over-methylated amines. The Mitsunobu–Fukuyama reaction was used for the conversion of primary amines to secondary mono-methylated amines in solution using an alcohol and via temporary protection of the amino group to avoid over methylation [17–21]. This method relies on the introduction of the *o*- or *p*-nitrobenzenesulfonyl groups to primary amines in the first step. The semi-protected sulfonamides can then undergo a selective mono-methylation via Mitsunobu reaction or by direct methylation. The reaction is completed by the selective removal of the sulfonamide group. Miller and Scanlan adjusted the *o*-NBS strategy to solid-phase synthesis and introduced a general three-step procedure for *N^α^*-mono-methylation of amino acids on solid support that was based on the work of Fukuyama [17,22–23]. Kessler and co-workers presented a time saving and cost effective three-step *N*-methylation procedure on solid support [3,24–25]. These improvements enabled, for the first time, the synthesis of a combinatorial library of all possible *N*-methylated analogues of a given sequence [26]. These strategies have been used over two decades as standard procedures for *N*-methylations of linear and cyclic peptides, proteins, and small molecules on solid support, in addition to other organic compounds in solution phase [22,24–25,27].

**Figure 1:**
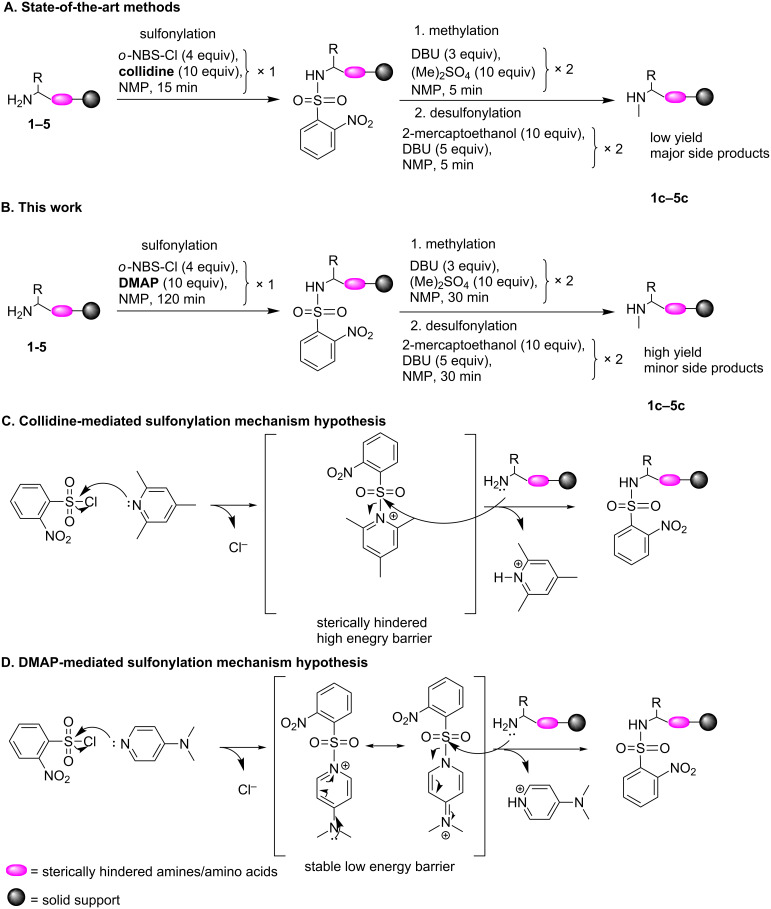
Collidine-assisted vs DMAP-assisted *N*-methylation process on solid support. (A) Collidine-assisted methylation of amines on solid support via a state-of-the-art method. (B) DMAP-assisted methylation of amine motifs on solid support as reported in this study. Comparative sulfonylation nucleophilic addition type mechanism as demonstrated for (C) collidine-mediated sulfonylation and (D) DMAP-mediated sulfonylation.

We found that the introduction of a single methyl group to an amine adjacent to a hindered moiety using the state-of-the-art strategies is extremely challenging (Figure 1A). Our study proved that the drop in yield of the entire *N*-methylation process is mostly due to an inefficient sulfonylation. We showed that the sulfonylation reaction of *o*-NBS-Cl using 2,4,6-collidine (collidine) as additive, which is commonly used for this synthetic transformation, is inefficient for several amine motifs. 4-Dimethylaminopyridine (DMAP) has been reported to assist in the sulfonylation and acylation of weak nucleophiles such as secondary amines, alcohols, amides, and sterically hindered amines when used as catalyst in addition to a base additive [28–31]. It has been suggested that these transformations proceed via a stable sulfonyl-DMAP intermediate [31–34]. Although many reported mechanistic studies reflect on the unique significance of the *para*-dialkylamino group [32] to the stabilization of the intermediate, DMAP was never reported to be used as the sole additive for sulfonylation of primary amines with *o*-NBS-Cl for the purpose of *N*-methylation on solid support.

We herein report a DMAP-mediated strategy for the efficient regioselective *N*-methylation of sterically hindered primary amines as well as less hindered amines on solid support (Figure 1B). Our DFT calculations provide an explanation to the observed superiority of DMAP over collidine as the lower energy barrier of the DMAP intermediate compared to that of the collidine intermediate is essential for a sulfonylation that proceeds through a nucleophilic addition type mechanism (Figure 1C and 1D). In addition to overcoming the setback associated with inefficient sulfonylation, we showed that other changes in the subsequent steps of the synthetic strategy can further improve the overall *N*-methylation process. This development enabled the methylation of various amine motifs on solid support and allowed for the synthesis of multiple-sites methylated peptide that could not be synthesized using the state-of-the-art strategies.

## Results and Discussion

**Figure 2:**
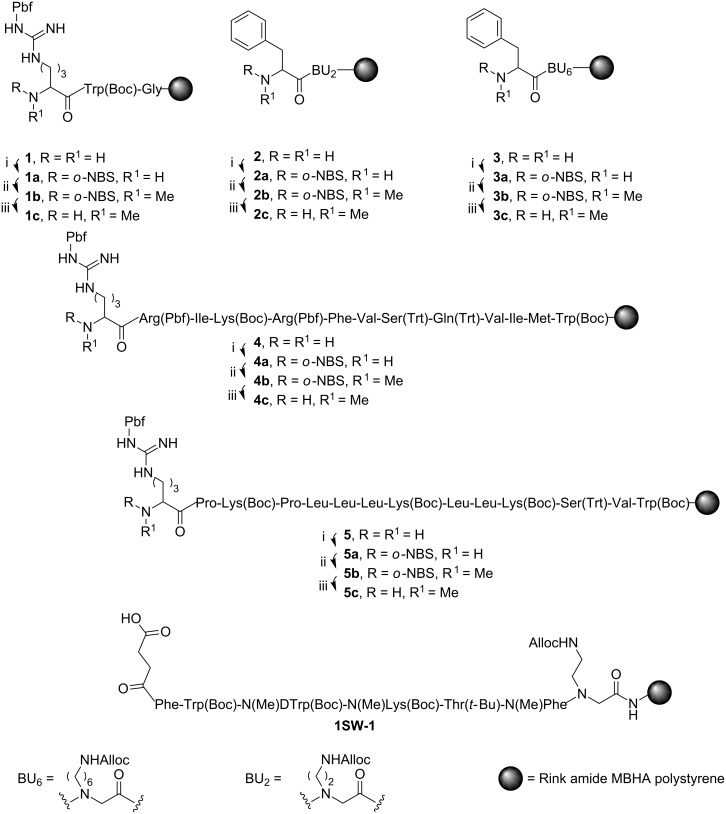
Motifs **1–5** were used as models for the optimization of the *N*-methylation process. i) Introduction of the *o*-NBS group to amines **1–5** using *o*-NBS-Cl. ii) Methylation of sulfonylamides **1a–5a** using (Me)_2_SO_4_. iii) Removal of the *o*-NBS group using 2-mercaptoethanol to give methylated **1c–5c**. **1SW-1** is a multiple sites *N*-methylated analogue of somatostatin.

The backbone precyclic somatostatin *N*^α^-methylated analogue, peptide **1SW-1** (Figure 2), possesses three methylation sites on three different amino acids in addition to a protected *N*^α^-alkylated glycine in the sequence. Because of its complexity, **1SW-1** was selected as our model in order to evaluate the efficiency of multiple sites *N*-methylation synthesis. The synthesis of **1SW-1** using the standard protocols yielded a crude mixture in which the isolation of the desired product was practically impossible. Various motifs with different sequences, sizes and chemical nature (Figure 2, **1–5**) in which some are related to **1SW-1** and others are known as highly hindered were used as models for estimating the influence of reaction conditions on the conversion yields. Attempts to synthesize the *N*-methylated motifs **1c–5c** using the state-of-the-art strategies resulted in low yields and highly contaminated crudes.

Our study revealed that the sharp drop in yields is associated with the inefficient methylation process of these amines. Detailed analysis indicated that the introduction of *o*-NBS to motifs **1**–**5** resulted in incomplete sulfonylation of the amine and led to the formation of many byproducts (see Table 1).

**Table 1:** Optimization of the sulfonylation step^a^.

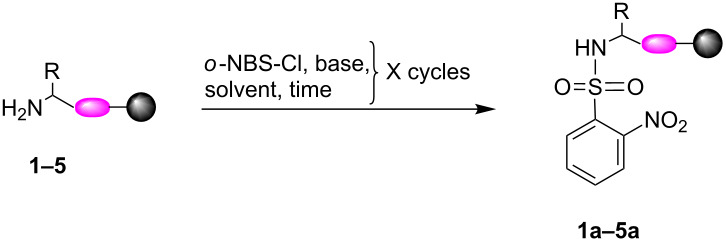							

entry	reactant	product	base	time (min)	cycle	conversion^b^	byproduct^c^

1	**1**	**1a**	collidine	15	1	57%	minor
2	**1**	**1a**	collidine	15	2	60%	minor
3	**1**	**1a**	collidine	60	2	73%	major
4	**1**	**1a**	collidine	120	2	76%	major
5^d^	**1**	**1a**	collidine	15	2	74%	major
6^d^	**1**	**1a**	collidine	O.N.^e^	1	86%	major
7	**1**	**1a**	DBU	120	1	N.R.^f^	–
8	**1**	**1a**	DMAP	120	1	98%	N.D.
9	**2**	**2a**	collidine	15	2	60%	major
10	**2**	**2a**	DMAP	120	1	100%	N.D.
11	**3**	**3a**	collidine	15	2	70%	major
12	**3**	**3a**	DMAP	120	1	100%	N.D.
13	**4**	**4a**	collidine	120	1	58%	minor
14	**4**	**4a**	DMAP	120	1	98%	N.D.
15	**4**	**4a**	DBU	120	1	N.R.^f^	–
16	**4**	**4a**	pyridine	120	1	48%	minor
17	**5**	**5a**	collidine	120	2	74%	minor
18	**5**	**5a**	DMAP	120	1	89%	N.D.

^a^General optimization conditions: **1–5** (0.35 mmol), *o*-NBS-Cl (4 equiv), base (10 equiv), NMP (5–10 mL), room temperature. ^b^Conversion to product, calculated based on: ((area under product peak)/(area under product peak + area under reactant peak)) × 100%, determined using HPLC. ^c^Byproduct: major = significant byproduct; minor = insignificant byproduct; N.D. = no byproduct. ^d^Higher equivalents of reagents were used. ^e^O.N. = overnight. ^f^N.R. = no reaction.

The efficiency of the *o*-NBS introduction to these motifs was evaluated. The most common strategies for sulfonylation of amines on solid support utilize a combination of *o*-NBS-Cl and collidine [22–25]. The HPLC results indicated that the conversion of motifs **1–5** to sulfonylamides **1a–5a** using these conditions never reached a conversion of above 74% (see Table 1, entries 2, 9, 11, 13, and 17). Moreover, the HPLC analysis indicated that a large quantity of impurities was accumulated already in this step.

Various reaction conditions were systematically changed to evaluate their effect on the conversion of amine **1** to sulfonylamide **1a**. Increasing the equivalents of the collidine reagent (Table 1, entries 5 and 6), changing the reaction solvent (Table 1, entry 5), repeating the reaction cycles (Table 1, entries 2–5), and increasing the reaction temperature (from room temperature to 55 °C, data not shown) all did not lead to a significant improvement in the conversion according to our HPLC results. Furthermore, the synthesis still resulted in the accumulation of side-products.

The effect of reaction time on conversion was evaluated by increasing the reaction time from 15 minutes to over 12 hours. HPLC analysis indicated that extending the incubation time to 120 minutes did result in an increase of the conversion but the reaction never went to completion and no significant decrease in the amount of side products was observed (Table 1, entries 3 and 4). When a combination of the most optimal conditions until this point was used, the conversion of **1** to **1a** reached only 86% (Table 1, entry 6).

Collidine is a common reagent for solid-phase synthesis (SPS) reagent and is applied as the additive of choice for the introduction of the *o*-NBS group in *N*-methylation protocols over the last two decades [22–25].

Collidine is a substituted pyridine-like additive, which acts as an efficient base. However, it is a weak nucleophile due to the high steric hindrance. Our results clearly show that collidine is an inadequate additive for sulfonylation and fall short of the conversion efficiency expected for a routinely used solid phase transformation (see above).

Sulfonylation of amines can proceed via two distinctive mechanisms. The first being a direct sulfonylation of the amine in which the additive acts as a base. The second being a nucleophilic addition type mechanism, in which a stable intermediate is formed between the sulfonyl and the additive.

We hypothesized that if the sulfonylation goes through a nucleophilic addition-type mechanism, replacing collidine with a more efficient nucleophilic additive would result in an overall improvement of the sulfonylation reaction and, subsequently, the entire *N*-methylation process. While if the dominant mechanism is a direct sulfonylation an improvement would be achieved simply using a more efficient base.

To challenge our hypothesis collidine was replaced by two SPS compatible bases namely, 1,8-diazabicyclo[5,4,0]undec-7-ene (DBU, p*K*_b_ ≈ 1.10) which is a stronger base than collidine (p*K*_b_ ≈ 6.57, Table 1, entry 7) and by DMAP (p*K*_b_ ≈ 4.48) which is a superior nucleophile (Table 1, entry 8).

HPLC analysis of the crude demonstrated that replacing collidine with DMAP resulted in a dramatic improvement in the conversion of **1** to **1a** (98%) and a significant decrease in the amount of the byproducts (Table 1, entry 8). On the other hand, replacing the collidine by DBU resulted in no conversion of **1** to **1a** (Table 1, entry 7).

**Figure 3:**
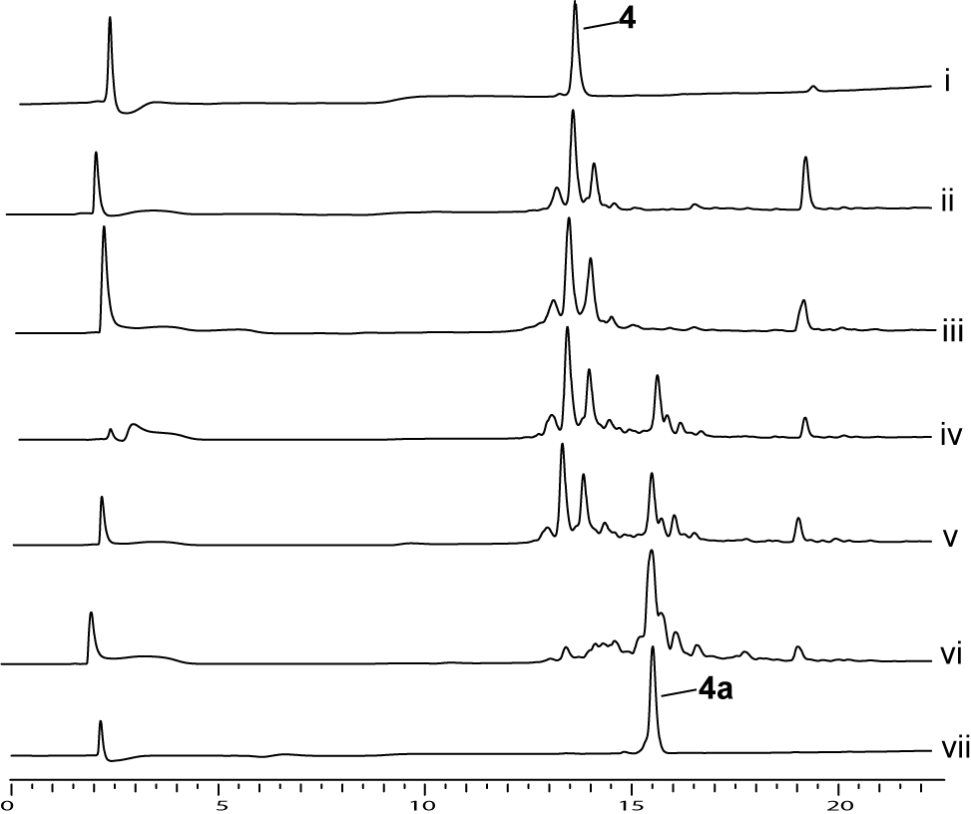
Sulfonylation optimization study. HPLC trace overlay that shows the sulfonylation of motif **4** to yield sulfonylamide **4a** under different reaction conditions. i) purified **4**. ii) crude **4**. vii) purified **4a**. HPLC trace of crude reaction mixture after 2 h treatment with *o*-NBS-Cl using the following additives: iii) DBU, iv) pyridine, v) collidine, and vi) DMAP.

To further evaluate the effect of base on the conversion and purity, another study was performed with motif **4** as a model using DBU, DMAP, pyridine, and collidine as additives. HPLC analysis showed that the additive used indeed has an impact on the conversion of **4** to **4a** and on the crude purity. DBU proved to be the poorest additive as no conversion was detected (Figure 3, iii). The analysis indicated that using collidine and pyridine as additives resulted in low conversion and in the accumulation of byproducts (Figure 3, iv and v). When DMAP was used, a conversion of 98% from motif **4** to motif **4a** was recorded with almost no byproducts (Figure 3, vi).

The replacement of collidine with the other additives provided us with important information. DBU, which is considered a more reactive base than the other reagents, proved the less effective additive. This suggested that the reaction is not governed by the basicity of the reagent. This was further confirmed by comparing the effect of pyridine to that of collidine. Pyridine (p*K*_b_ ≈ 8.80) and collidine (p*K*_b_ ≈ 6.57) are reagents that share many similar features with the later considered as a more reactive base due to the multiple electron-donating groups on the aromatic ring. Nevertheless, our study indicated that both pyridine and collidine had almost the same effect on the conversion. Both observations imply that the mechanism involved in the introduction of the *o*-NBS group to the amine is not related to the basicity of the reagent used.

DMAP plays a wide catalytic role in various synthetic transformations, e.g., acylations, alkylations, silylations, esterifications, and many others, as it acts as nucleophile rather than a base [33–40]. Compared to DMAP, pyridine is a weak nucleophile as it does not have any electron-donating groups on the ring. Collidine has electron-donating groups that make it nucleophilic but it is sterically hindered, while DMAP is stabilized by resonance and much less hindered.

Our results emphasized the effect of DMAP for the introduction of the *o*-NBS group as it proved the most effective reagent compared to the other additives used. DMAP is used as a catalyst in acylation reactions as it acts via a nucleophilic addition mechanism [34,37,40]. In nucleophilic catalysed acylation, the mechanism involves a step in which the DMAP replaces the leaving group on the carbonyl group forming a stable reactive intermediate that lowers the overall energy barrier. This stabilization effect is unique to *para*-dialkylaminopyridine derivatives and much less significant for other pyridine type analogues like collidine.

We suggested that the significant effect of DMAP compared to that of collidine on the sulfonylation reaction with *o*-NBS-Cl might be explained by the formation of a low energy sulfonyl-DMAP intermediate that is the typical species that dominates a catalytic nucleophilic addition mechanism.

**Figure 4:**
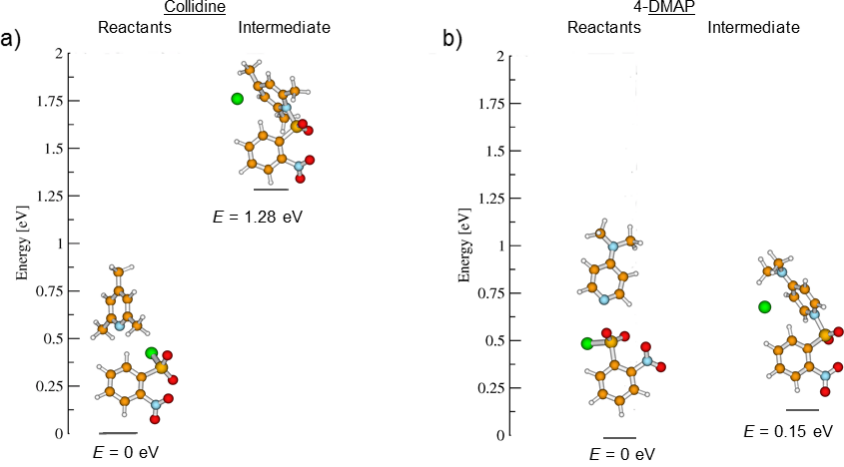
DFT calculations for the reaction of *o*-NBS-Cl with a) collidine and b) DMAP. The structure of the reactants and the energies calculated appear on the left while the structure and calculated energies of the intermediates appear on the right of each column.

To explore the possibility that the sulfonylation step takes place in a catalytic nucleophilic addition type mechanism, a DFT study was performed. In this study, the effect of collidine and DMAP on the formation of sulfonylamide was evaluated by calculating the energy differences between the reactants and their corresponding intermediates (Figure 4). The calculations include the chlorine ion that contributes to the stabilization of the positive charge of the intermediate [41–44]. Figure 4a compares the energy of the *o*-NBS and the collidine reactants to the energy of the corresponding intermediate.

As can be seen, the energy difference between both structures is quite large, namely 1.28 eV. Figure 4b shows the energy diagram for the reaction of *o*-NBS-Cl with DMAP. The energy difference in this case is much lower, namely only 0.15 eV. The DFT study shows that the energy barrier of the *o*-NBS-DMAP intermediate is low compared to that of the *o*-NBS-collidine intermediate. These results confirm that the difference in reactivity is related to the low energy barrier of the *o*-NBS-DMAP intermediate compared to that of collidine and that the sulfonylation of the amine takes place in a nucleophilic addition type mechanism which benefits dramatically from the unique nucleophilic nature of DMAP (Figure 1D).

In the first part of the suggested mechanism, the pyridine base substitutes the chloride to form a sulfonylpyridinium intermediate. This intermediate makes the sulfonyl group a better electrophile, hence, the attack of the primary amine in the second part of the mechanism becomes more feasible. DMAP forms a more stable intermediate compared to collidine. DMAP is unique since the lone pair electrons of the tertiary dimethylamine can stabilize the overall charge by resonance (Figure 1D). On the other hand, collidine is sterically hindered and does not have a resonance stabilization group. These two facts explain the main difference between collidine and DMAP. This analysis suggests that the relatively high energy of the *o*-NBS-collidine intermediate is the major cause for the inefficiency of the sulfonylation using this additive.

The studies performed on motifs **1** and **4** suggested that a combination of extending the reaction time to 120 minutes and, most importantly, replacing the collidine with DMAP can dramatically improve the reaction efficiency (see Supporting Information File 1 for the complete detailed procedure). This was confirmed also for motifs **2**, **3** and **5** (Table 1).

The use of DMAP as catalyst to enhance sulfonylation of amines, amides and alcohol has been previously described (see above). In the reports describing sulfonylation of amines, DMAP was added to a reaction mixture that contains a large excess of tertiary base or pyridine. Our study proves that the use of bases like pyridine, Et_3_N or Hünig’s base is not at all required for this transformation as DMAP itself can be used most efficiently without any additional base. Moreover, DMAP has been reported most extensively for the sulfonylation of weak nucleophiles like alcohols as it provides a very reactive intermediate. We proved that although it is completely unexpected for primary amines, the case here is very similar to alcohols and that the formation of the stable DMAP-SO_2_R intermediate is crucial for this transformation. Our calculations clearly indicate that collidine does not produce an intermediate with the proper stability to catalyse the reaction and, hence, fall short of delivering satisfying results. Our comparative study proves that the basic-conditions-derived mechanism is not the preferred mechanism for sulfonylation of primary amines and suggests that the exclusive use of DMAP is sufficient. The use of DMAP for the introduction of the *o*-NBS group is extremely important in the context of *N*-methylation, since *o*-NBS introduction as protecting/activating group is essential to the entire process.

In order to further establish our method we optimized the next two steps in the process to provide a fully efficient methylation method of hindered systems. The sulfonylamides **1a–5a** were methylated to give the corresponding *N*-methylated sulfonylamides **1b–5b** and the *o*-NBS group was later removed from the methylated sulfonylamides **2b, 3b** and **5b** to yield the final *N*-methylated motifs.

The methylation was performed by incubation of sulfonylamides **1a–5a** with (Me)_2_SO_4_ in the presence of DBU for five minutes according to the reported procedure [25]. These trials resulted in insufficient conversion (Table 2, entries 1, 5, 7, 10, and 13). Sulfonylamide motifs **1a–5a**, were then methylated using different conditions.

**Table 2:** Optimization of the *N*-methylation reaction (step ii)^a^.

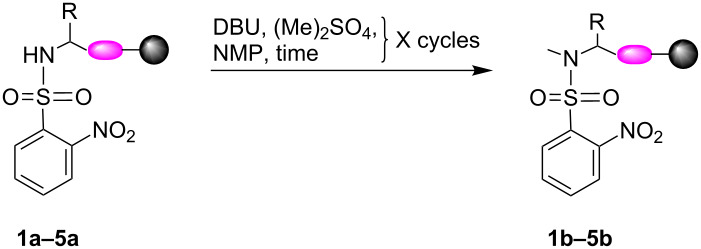					

entry	reactant	product	time (min)	cycle	conversion^b^

1	**1a**	**1b**	2	2	10%
2	**1a**	**1b**	30	1	22%
3^c^	**1a**	**1b**	30	2	N.R.^d^
4	**1a**	**1b**	30	2	40%
5	**2a**	**2b**	2	2	47%
6	**2a**	**2b**	30	2	93%
7	**3a**	**3b**	2	2	40%
8	**3a**	**3b**	30	1	88%
9	**3a**	**3b**	30	2	99%
10	**4a**	**4b**	2	2	60%
11	**4a**	**4b**	30	1	65%
12	**4a**	**4b**	30	2	80%
13	**5a**	**5b**	2	2	84%
14	**5a**	**5b**	30	2	96%

^a^General optimization conditions: **1a–5a** (0.35 mmol), DBU (3 equiv), NMP (5–10 mL), pre-activation 3 min, addition of (Me)_2_SO_4_ (10 equiv), room temperature. ^b^Conversion to product, calculated based on: ((area under product peak)/(area under product peak + area under reactant peak)) × 100%, determined by HPLC. ^c^DMAP was used as base. ^d^N.R. no reaction.

Few parameters were changed systematically to evaluate their effect on the conversion and compared to the best known state-of-the-art conditions (Table 2).

**Figure 5:**
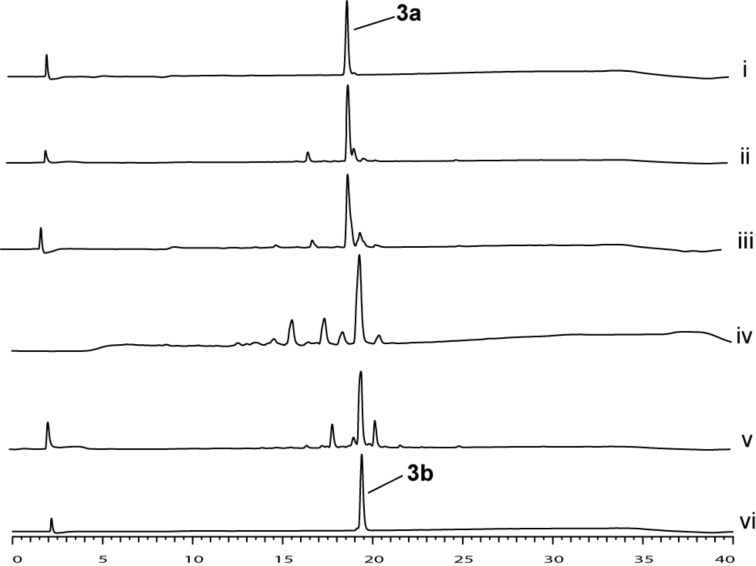
Methylation of motif **3a** to **3b** using various reaction conditions. HPLC trace overlay presents the effect of different reaction conditions on the conversion yield. HPLC trace of i) purified motif **3a**. ii) crude motif **3a**. vi) purified methylated product **3b**. *N*-methylation reaction using: iii) two cycles of 5 min; iv) one cycle of 30 min; v) two cycles of 30 min each.

In all cases, increasing the incubation time to 30 minutes and repeating the reaction twice resulted in a significant improvement in the conversion yield (Table 2, Figure 5 and Supporting Information File 1). The effect is demonstrated for motif **3** as the conversion was increased from 40% (Figure 5, iii) to 99% (Figure 5, v) by using the improved conditions.

The conversion of **1a** to **1b** did not go to completion. It might be attributed to a specific conformation that makes **1a** less accessible to methylation compared to the other models. However, the improved method provided the best conversion from **1a** to **1b** compared to the other methods used (Table 2, entries 1–4).

To complete the full methylation process, the *o*-NBS removal step was also addressed. The *o*-NBS removal from motifs **2b**, **3b**, and **5b** was performed using a combination of 2-mercaptoethanol and DBU according to previously reported procedures. When the reaction was incubated for 5 minutes at room temperature the conversion was very low, especially for **2b** and **5b** (Table 3, entries 1, 3, and 6). A procedure using 30 minutes incubation time was tested. For motifs **2c**, **3c**, and **5c** the conversion using the extended incubation time resulted in almost complete removal of the *o*-NBS to yield the methylated secondary amines (Table 3, entries 2, 5, and 7).

**Table 3:** Optimization of the *o*-NBS removal reaction (step iii)^a^.

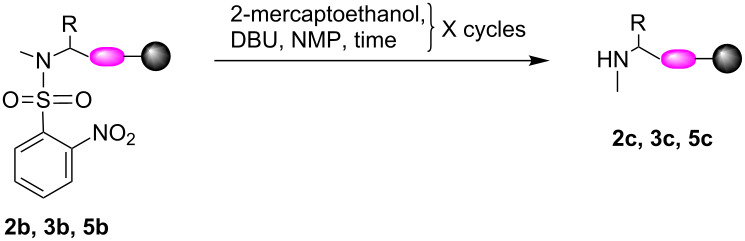					

entry	reactant	product	time (min)	cycle	conversion^b^

1	**2b**	**2c**	5	2	10%
2	**2b**	**2c**	30	2	98%
3	**3b**	**3c**	5	2	80%
4*^c^*	**3b**	**3c**	5	2	80%
5	**3b**	**3c**	30	1	98%
6	**5b**	**5c**	5	2	16%
7	**5b**	**5c**	30	2	100%

^a^General optimization conditions: **2b**, **3b**, **5b** (0.35 mmol), DBU (5 equiv), 2-mercaptoethanol (10 equiv), NMP (5–10 mL), room temperature. ^b^Conversion to product, calculated based on: ((area under product peak)/(area under product peak + area under reactant peak)) × 100%, determined using HPLC. ^c^Higher equivalents of reagents were used.

**Figure 6:**
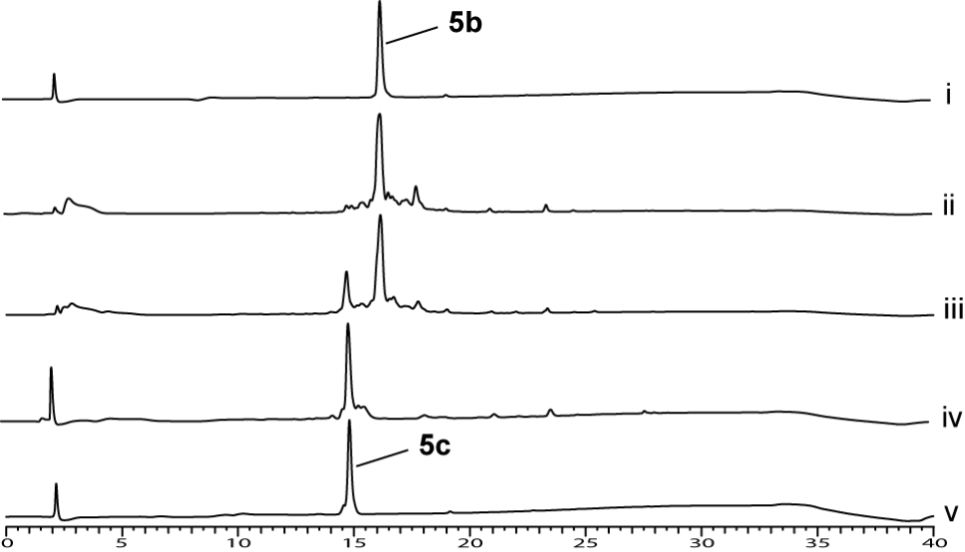
Optimization of *o*-NBS removal reaction conditions demonstrated on motif **5b**. HPLC trace overlay of i) purified motif **5b**. v) purified motif **5c**. ii) Crude reactant **5b**. HPLC trace of crude mixture after removal of the *o*-NBS group using either, iii) two cycles of 5 minutes incubation or iv) two cycles of 30 minutes incubation.

An HPLC trace overlay for the conversion of motif **5b** to motif **5c** is presented to demonstrate the difference in efficiency (Figure 6). This study showed that the incubation time was crucial for the completion of the *o*-NBS removal.

A full schematic description of the method is presented in Figure 1B (see also the detailed procedure in Supporting Information File 1). After completing the new procedure the methylated amine can be coupled with an amino acid to form a methylated amide. This procedure was, hence, suitable for the synthesis of peptides with multiple sites methylated amides.

To further evaluate the efficiency of the new methylation method, the SPS of the multi-sites methylated peptide **1SW-1** was re-attempted by using our method and was compared to the synthesis using the state-of-the-art methylation method.

**Figure 7:**
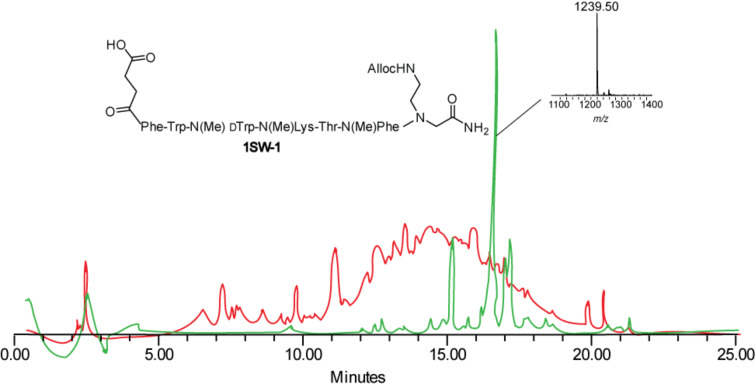
HPLC trace overlay and MS analysis of the somatostatin analogue, **1SW-1**, which was *N*^α^-methylated on solid support at three different sites. Red: *N*^α^-methylations were performed using the state-of-the art procedure. Green: *N*^α^-methylations were performed using the DMAP-assisted procedure reported here.

We revealed that while isolation of the desired product was not even possible after using the state-of-the-art methylation collidine based protocols (Figure 7 red), peptide **1SW-1** could be easily isolated after using the method reported herein (Figure 7 green). This study proved that our strategy can be efficiently applied for the sequential *N*^α^-methylation of different amino acids in a regioselective manner.

## Conclusion

Synthetic *N*-methylated compounds are extremely important tools that find applications in many fields. This study presents an improved three-step SPS *N*-methylation strategy that allowed accessibility to single and multi-site methylated compounds, which could not be synthesized using the state-of-the-art methods. This work highlights the essential factors for achieving an efficient regioselective methylation of primary amines and focused mostly on the sulfonylation step as it proved the Achilles heel of the common strategies. This work proved that collidine, the most widely used sulfonylation additive in *N*-methylation related strategies, is far from being optimal for this transformation. DMAP proved superior to collidine as a reagent for the sulfonylation of primary amines on solid support. Our study emphasizes that sulfonylation can proceed using DMAP as a single additive. We claim that the presented strategy will enable the synthesis of many, otherwise privileged, *N*-methylated compounds, hence, will have a major impact on the related fields.

## Supporting Information

File 1 Experimental part, synthetic procedures, solid-phase synthesis protocols, HPLC chromatograms, mass spectrometry analysis.
