# Supplementary material for: DMAP-assisted sulfonylation as an efficient step for the methylation of primary amine motifs on solid support
Source: Beilstein J Org Chem. 2017 May 3;13:806–16. doi: 10.3762/bjoc.13.81 (PMC5433225; doi:10.3762/bjoc.13.81)
Supplement: File 1 — Experimental part, synthetic procedures, solid-phase synthesis protocols, HPLC chromatograms, mass spectrometry analysis. [file Beilstein_J_Org_Chem-13-806-s001.pdf]

Supporting information

for

DMAP-assisted sulfonylation as an efficient step for the methylation of primary amine motifs on solid support

Johnny N. Naoum[1], Koushik Chandra[1,§], Dorit Shemesh[1, 2], R. Benny Gerber[1, 2], Chaim Gilon[1], and Mattan Hurevich*[1]

Address: [1]Institute of Chemistry, The Hebrew University of Jerusalem, Edmond Safra Campus, Givat Ram, Jerusalem, 91904, Israel and [2]Fritz Haber Research Center, The Hebrew University of Jerusalem, Jerusalem 91904, Israel

[§]Current address: Department of Chemistry, Midnapore College (Autonomous), Raja Bazar Main Road, Paschim Medinipur, West Bengal, India

Email: Mattan Hurevich* - mattan.hurevich@mail.huji.ac.il

* Corresponding author

Experimental part, synthetic procedures, solid-phase synthesis protocols, HPLC chromatograms, mass spectrometry analysis

Contents

1.0 Experimental section: S3

1.1 Materials S3

1.2 General methods S3

1.3 Solid phase synthesis S4

1.3.1 Coupling protocols: S4

1.3.2 Complete three steps *N*-methylation strategy: S5

1.3.3 Synthesis of Alloc *N*-alkylated glycine building units on solid support S6

1.3.4 Synthesis of **1SW-1** S7

1.3.5 Cleavage from solid support and work-up S7
1.3.6 The procedure of the small cleavage: S8
1.4 DFT calculations: S8
2.0 RP-HPLC chromatograms recorded after each reaction step for the different motifs applying various reaction conditions S9
2.1 HPLC chromatograms of the sulfonylation step S9
2.1.1 Sulfonylation studies of **1** to get **1a** S9
2.1.2 Sulfonylation studies of **2** to get **2a** S16
2.1.3 Sulfonylation studies of **3** to get **3a** S18
2.1.4 Sulfonylation studies of **4** to get **4a** S19
2.1.5 Sulfonylation studies of **5** to get **5a** S20
2.2 HPLC chromatograms of the crude motifs after the methylation step S22
2.2.1 *N*-methylation of **1a** to get **1b** S22
2.2.2 *N*-methylation of **2a** to get **2b** S26
2.2.3 *N*-methylation of **3a** to get **3b** S27
2.2.4 *N*-methylation of **4a** to get **4b** S28
2.2.5 *N*-methylation of **5a** to get **5b** S31
2.3 HPLC chromatograms of the crude motifs after the *o*-NBS removal step S33
2.3.1 *o*-NBS removal from **2b** to get **2c** S33
2.3.2 *o*-NBS removal from **3b** to get **3c** S35
2.3.3 *o*-NBS removal from **5b** to get **5c** S37
2.4 HPLC chromatograms and HRMS characterization of **1SW-1** S38
2.4.1 HPLC chromatogram of the crude peptide **1SW-1** synthesized via standard method S38
2.4.2 HPLC chromatogram of the crude peptide **1SW-1** synthesized following the improved new SPS *N*-methylation method S39
2.4.3 HRMS analysis of isolated **1SW-1** synthesized following the improved new SPS *N*-methylation method S39
3.0 Tables S40
3.1 Characterization and detailed reaction conditions of the sulfonylation step S40
3.2 Characterization and detailed reaction conditions of the methylation step S42
3.3 Characterization and detailed reaction conditions of the *o*-NBS removal step S44

1.0 Experimental section:

1.1 Materials

9-Fluorenylmethyloxycarbonyl (Fmoc)-N^{α}-protected amino acids, O-(7-azabenzotriazol-1-yl)-N,N,N′,N′-tetramethyluronium hexafluorophosphate (HATU), 1-hydroxy-7-azabenzotriazole (HOAt), were purchased form Chem-Impex International Inc (Wood Dale, IL, U.S.A.). BTC, $(Me)_2SO_4$, DBU, 2,4,6-collidine, *o*-NBS-Cl, 2-mercaptoethanol, MeOH, TFA, diethyl ether, piperidine, DIPEA, 1,2-diaminoethane, 1,6-diaminohexane, 4-dimethylaminopyridine (DMAP) and other organic materials were purchased from ACROS (Geel, Belgium). Fmoc-Rink-Amide methylbenzhydrylamine (MBHA) resin (200–400 mesh, 0.53 and 0.71 mmol/g resin) was purchased from Iris Biotech GMBH (Marktredwitz, Germany). Organic solvents for solid phase synthesis and HPLC: NMP, DCM, DMF, and ACN were purchased from J.T.Baker (NJ, USA).

1.2 General methods

Mass spectra were obtained using Voyager-DE PRO Biospectrometry workstation using matrix assisted laser desorption ionization-time of flight (MALDI-TOF) technology in the positive mode or electrospray ionization MS was performed on LCQ Fleet Ion Trap mass spectrometer (Thermo Scientific). HRMS were recorded on Agilent 6550 iFunnel Q-TOF LC/MS system.

Analytical HPLC were performed using a Vydac analytical RP column (C18, 4.6 × 250 mm, 5 µm) and was carried out on a Merck-Hitachi L-7100 pump and a Merck-Hitachi L-7400 variable wavelength detector operating at 215–220 nm. The mobile phase consisted of a gradient system that included solvents A and B referring to TDW (0.1% TFA) and ACN (0.085% TFA), respectively. The mobile phase started with 95% A from 0 to 5 minutes followed by linear gradient from 5% B to 95% B from 5 to 25 minutes. The gradient remained at 95% B for 5 minutes and then was reduced back to the starting conditions within 5 minutes. The gradient remained at 95% A for additional 5 minutes for column equilibration. The flow rate of the mobile phase was 1 mL/min.

Motifs and peptides purification was performed by reversed phase HPLC (RP-HPLC) (on L-6200A pump, Merck-Hitachi, Japan), using a Vydac preparative RP column (C18, 22 ×

250 mm, 10 µm). The mobile phase consisted of a gradient system that included solvents A and B referring to TDW (0.1% TFA) and ACN (0.085% TFA), respectively. The mobile phase started with 95% A from 0 to 5 minutes followed by linear gradient from 5% B to 95% B from 5 to 45 minutes. The gradient remained at 95% B for 5 minutes, and then was reduced back to the starting conditions within 5 minutes. The gradient remained at 95% A for additional 5 minutes for column equilibration. The flow rate of the mobile phase was 9 mL/min. These HPLC gradient plans and conditions were sufficient concerning our research sequences.

1.3 Solid phase synthesis

In general, the synthesis was performed either in a 25 mL sep-pack or in a reaction vessel equipped with a sintered glass bottom applying general Fmoc chemistry protocols for solid phase synthesis (SPS): Fmoc rink amide methylbenzhydrylamine (Fmoc-MBHA) resin (0.5 g, loading 0.53 or 0.71 mmol/g·resin) was pre-swollen in NMP for 3 hours. The equivalents of all reagents were in respect to the resin weight and loading capacity, and the volume of the SPS solvent used in most of the reactions was fixed to 7–10 mL.

Capping was carried out after the anchoring of the first amino acid to the resin and performed via the new developed method of acetylation reported recently.[1]

Fmoc deprotection protocol: A solution of 20–25% piperidine in NMP was added to the reaction vessel. The reaction vessel was shaken for 30 minutes at room temperature after which the solvents were filtered and a new solution of 20–25% piperidine in NMP was added and shaken for another 30 minutes. Reaction solvents were drained out of the reaction vessels and the resin was washed four times with 10 mL of NMP and twice with 10 mL of DCM.

<u>1.3.1 Coupling protocols:</u>

Coupling of Fmoc-[Amino Acid]-OH was performed using one of two protocols described below:

Coupling protocol l (HATU): Fmoc-[Amino Acid]-OH (3 equiv) was dissolved in NMP in addition to HOAt (3 equiv), then HATU (3 equiv) was added to the solution for pre-activation and shaken in an ice bath (3 min). The pre-cooled pre-activated solution was

poured onto the resin and shaken (60 min) followed by washing with NMP (4 × 2 min) and DCM (2 × 2 min). The protocol was repeated twice to ensure reaction completion.

Coupling protocol 2 (BTC): Fmoc-[Amino Acid]-OH (3 equiv) was dissolved in DCM in addition to BTC (1 equiv), the solution was pre-cooled in an ice bath (3 min). 2,4,6-Collidine (20 equiv) was then added to the solution which was further cooled and shaken for only one minute (the reaction with BTC is vigorous). The pre-cooled pre-activated solution was poured onto the resin and shaken (60 min) followed by washing with DCM (5 × 2 min.). The protocol was repeated twice to ensure reaction completion.

The standard volume is necessary for dissolving all reagents, but more important for keeping a minimal volume in order to enable the reaction solution to be shaken properly, otherwise, part of the resins beads might stick to the reaction vessel/sep-pack wall and poorly react. From our laboratory experience, coupling via any of these two specific methods is considered excellent, for difficult as well as regular couplings, enabling driving reactions to completion in SPS. Besides, these coupling methods proceed very fast minimizing the odds for the competing slower racemization reactions. For difficult couplings, when some heating took place, the synthesis had to proceed in a sintered glass vessel that has water inlet and outlet for heating via water bath. The reactions mostly took place at room temperature, but in few cases the reaction vessel was heated up to 50–60 °C.

A cycle of Fmoc removal and coupling of Fmoc-AA-OH (protected at side chains if necessary) specific for the planned sequence took place until we matched the targeted motifs. The final motif-resin was washed thoroughly with DCM (5 × 2 min), MeOH (2 × 2 min) and dried under vacuum for (60 min). The motif-resin was further dried overnight in a desiccator.

1.3.2 Complete three steps *N*-methylation strategy developed in this work:

Step 1: A solution of *o*-NBS-Cl (4 equiv) and DMAP (10 equiv) in NMP (5 mL) was mixed for 1 minute before being added to **1–5** motif resins. The mixture was shaken for

120 minutes at room temperature. The reaction mixture was removed by filtration and the solid support was subsequently washed with NMP.

Step 2: A solution of DBU (3 equiv) in NMP (5 mL) was added to **1a**–**5a** motif-resins and shaken for 3 minutes before $(Me)_2SO_4$ (10 equiv) was added to the reaction solution. The reaction was shaken for 30 minutes at room temperature. The reaction solution was filtered and the resin was washed with NMP. This step was repeated twice for each motif.

Step 3: A solution of 2-mercaptoethanol (10 equiv) and DBU (5 equiv) in NMP (5 mL) was mixed for 1 minute and transferred to the motif resin which was pre-swollen in NMP. The reaction was incubated for 30 minutes at room temperature. The reaction solution was removed by filtration and the resin was washed with NMP. This step was repeated twice for each motif.

1.3.3 Synthesis of Alloc *N*-alkylated glycine building units on solid support

A solid phase anchored amine was pre-swollen with DMF. The resin was added a solution of diisopropylcarbodiimide (10 equiv) and bromoacetic acid (10 equiv) in 10 mL DMF that was pre-activated for 20 minutes. The mixture was shaken for 30 minutes at room temperature before solution was filtered and washed once with DMF (1 × 2 min). The procedure was repeated twice before the resin was extensively washed with DMF (3 × 2 min), DCM (2 × 2 min). The bromo functionalized solid support was treated with a solution of 10 equivalents of either Alloc-NH-$(CH_2)_2$-NH_2 or Alloc-NH-$(CH_2)_6$-NH_2 (prepared according to previous reports[2]) and DIPEA (20 equiv) in a total volume of 10 mL DMF. The mixture was shaken at 55 °C overnight before the solution was filtered and resin was washed with DMF (3 × 2 min), and DCM (2 × 2 min), dried in vacuum for 60 minutes and kept in a dissector overnight. Small cleavage and MS analysis were performed to confirm the mass of the synthesized units.

1.3.4 Synthesis of **1SW-1**: Alloc *N* alkylated glycine building unit 2 was prepared and anchored to the resin as described above. Fmoc-Phe-OH was coupled to the resin using the BTC coupling protocol. The Fmoc protecting group was removed and the free amine was methylated using the protocol described in section 1.3.2. Fmoc-Thr(*t*-Bu)-OH and Fmoc-Lys(Boc)-OH were subsequently coupled using HATU protocol. The Fmoc protecting group was removed and the free amine was methylated using the protocol described in section 1.3.2. Fmoc-D-Trp(Boc)-OH was coupled to the methylated amine using HATU protocol. Fmoc protecting group was removed and the free amine was methylated using the protocol described in section 1.3.2. Fmoc-Trp(Boc)-OH was coupled to the methylated amine using HATU protocol. The Fmoc protecting group was removed and Fmoc-Phe-OH was coupled using HATU protocol. The Fmoc protecting group was removed and the resin was incubated overnight with a solution of 10 equiv succinic anhydride and 1 equiv DMAP in DMF.

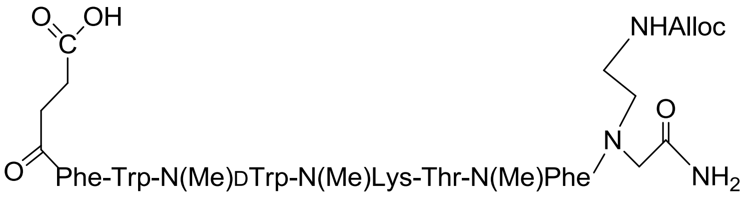

1SW-1

1.3.5 Cleavage from solid support and work-up: Cleavage from the resin and removal of side chain protecting groups were carried out simultaneously using a pre-cooled standard 10 ml cocktail composed of 95% TFA, 2.5% TDW, and 2.5% TIS added to about 0.7 g motif-resin. The mixture was kept standing for 30 minutes in an ice bath, and then was shaken for another 150 minutes at room temperature. The TFA filtrate (with the separated motif dissolved in) was partially evaporated by a stream of nitrogen, and cold diethyl ether or a cold mixture of diethyl ether/hexane (1:1) was added to the remained solution to remove the scavengers and other hydrophobic impurities, while the motif was precipitated by centrifugation. Diethyl ether or diethyl ether/hexane (1:1) was then removed by decanting. The precipitation and decanting was repeated three times. The dry crude motif was dissolved in ACN/TDW (1:1) and lyophilized overnight. The product

was usually obtained as fluffy white solid crude. Color tests of chloranil and Kaiser[3-5] indicated, with a good correlation to mass spectrometry results, wither the coupling or deprotection reactions took place. However, it was not reliable in all cases, especially when secondary amines were involved as in the *N*-methylation steps for instance. Therefore, small scale cleavage for HPLC/MS analysis were performed following every important step in the synthesis including the *N*-methylations. We named these evaluating points as the “checkpoints”.

<u>1.3.6 The procedure of the small cleavage:</u> A small amount of the motif-resin (few visible beads) was treated with a pre-cooled mixture of TFA (2 mL), TDW (1 drop) and TIS (1 drop) for 30 minutes in an Eppendorf. The separated resin beads were removed by filtration. TFA was fully evaporated with a stream of nitrogen. The residual was dissolved in an ACN: TDW (1:1) and examined by HPLC/MS.

1.4 DFT calculations:

DFT calculations using the B3-LYP functional with the cc-pVDZ basis set were performed on the *o*-NBS-Cl and collidine or the *o*-NBS-Cl and DMAP reactants and for the *o*-NBS-collidine or the *o*-NBS-DMAP intermediates after the substitution of the chlorine atom.[6-9] The chlorine ion after substitution was included in the calculation, since it has a stabilizing effect on the positive charged intermediate. All the structures were minimized. Harmonic frequencies were computed in order to ensure that all structures are indeed minima. The calculations were performed using the Turbomole program suite.[6] The predicted energy difference between the *o*-NBS-DMAP intermediate and the corresponding reactants is 0.15 eV, much lower than for the *o*-NBS-collidine intermediate and the corresponding reactants (1.28 eV).

2.0 RP-HPLC chromatograms recorded after each reaction step for the different motifs applying various reaction conditions

2.1 HPLC chromatograms of the sulfonylation step

In all cases, the crude motifs were removed from solid support after the reaction using either full or small cleavage protocols. The motifs were dissolved in TDW/ACN 1:1 mixture, filtered through a 0.45 μm PTFE filters and injected to an analytical RP-C18-HPLC without any purification.

<u>2.1.1 Sulfonylation studies of **1** to get **1a**</u>

<u>Sulfonylation of **1** using conditions described in Table 1 entry 1:</u>

Resin bound motif **1** was sulfonylated to **1a** following the conditions detailed in Table 1 entry 1.

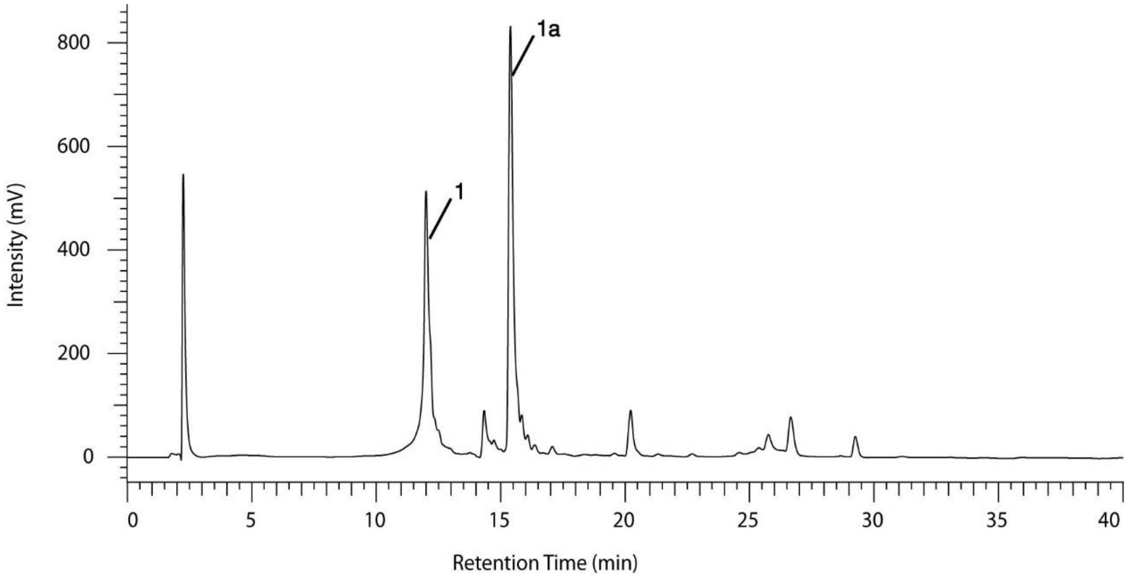

Figure S1. RP-HPLC Chromatogram of crude obtained after sulfonylation of **1** under the conditions described in Table 1 entry 1. (**conditions**: flow rate: 1 mL/min, Vydac analytical RP column (C18, 4.6 × 250 mm, 5 μm), recorded at 220 nm).

Sulfonylation of **1** using conditions described in Table 1 entry 2:

Resin bound motif **1** was sulfonylated to **1a** following the conditions detailed in Table 1 entry 2.

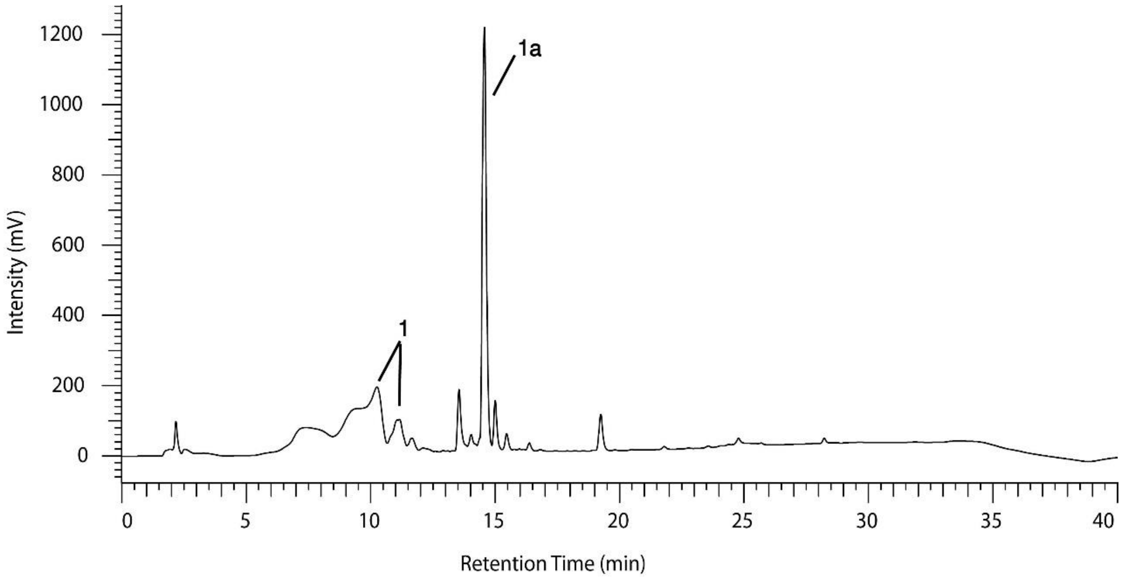

Figure S2. RP-HPLC Chromatogram of crude obtained after sulfonylation of **1** under the conditions described in Table 1 entry 2. (**conditions**: flow rate: 1 mL/min, Vydac analytical RP column (C18, 4.6 × 250 mm, 5 μm), recorded at 220 nm). Mass of motif **1** was observed in both peaks pointed above between 10 and 11 minutes.

Sulfonylation of **1** using conditions described in Table 1 entry 3:

Resin bound motif **1** was sulfonylated to **1a** following the conditions detailed in Table 1 entry 3.

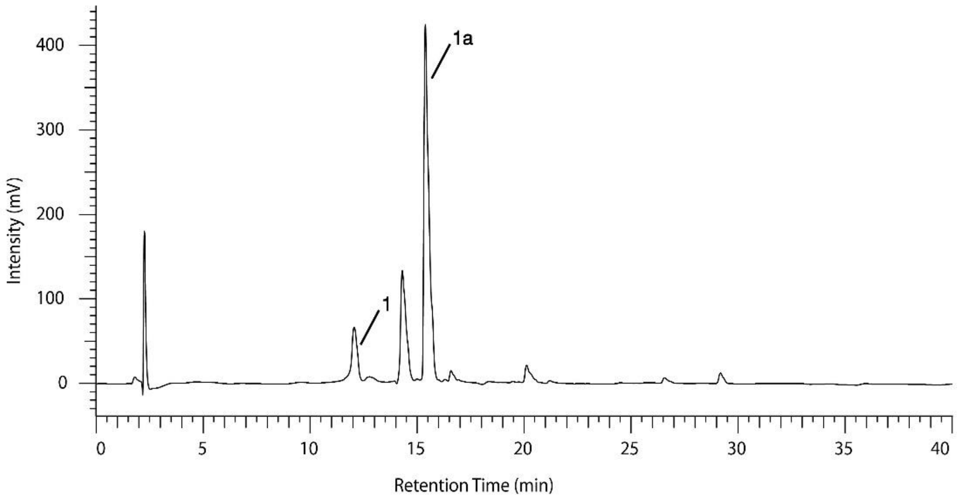

Figure S3. RP-HPLC Chromatogram of crude obtained after sulfonylation of **1** under the conditions described in Table 1 entry 3. (**conditions**: flow rate: 1 mL/min, Vydac analytical RP column (C18, 4.6 × 250 mm, 5 μm), recorded at 220 nm).

Sulfonylation of **1** using conditions described in Table 1 entry 4:

Resin bound motif **1** was sulfonylated to **1a** following the conditions detailed in Table 1 entry 4.

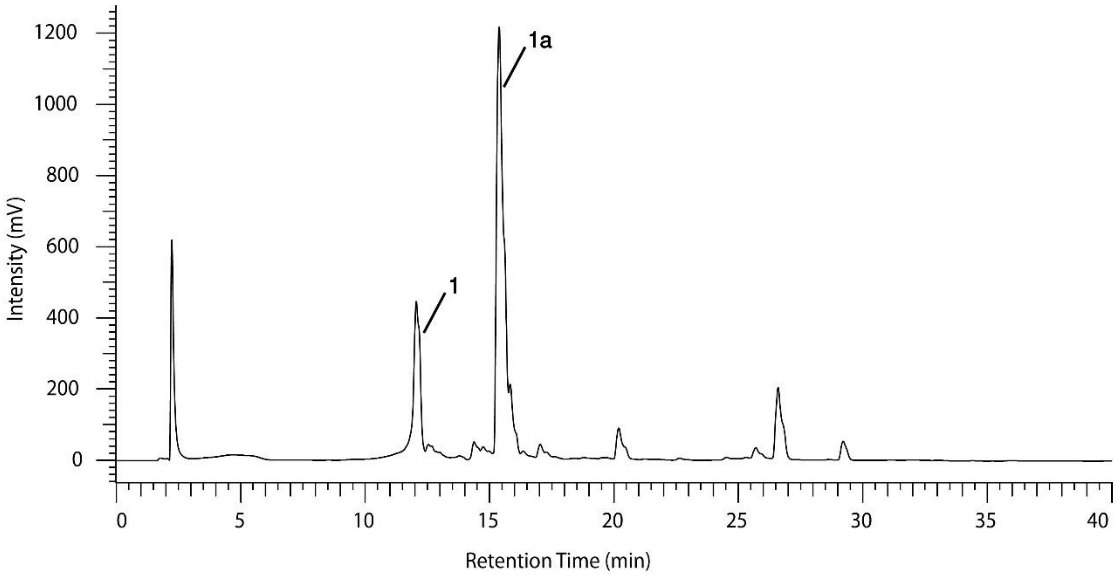

Figure S4. RP-HPLC Chromatogram of crude obtained after sulfonylation of **1** under the conditions described in Table 1 entry 4. (**conditions**: flow rate: 1 mL/min, Vydac analytical RP column (C18, 4.6 × 250 mm, 5 μm), recorded at 220 nm).

Sulfonylation of **1** using conditions described in Table 1 entry 5:

Resin bound motif **1** was sulfonylated to **1a** following the conditions detailed in Table 1 entry 5.

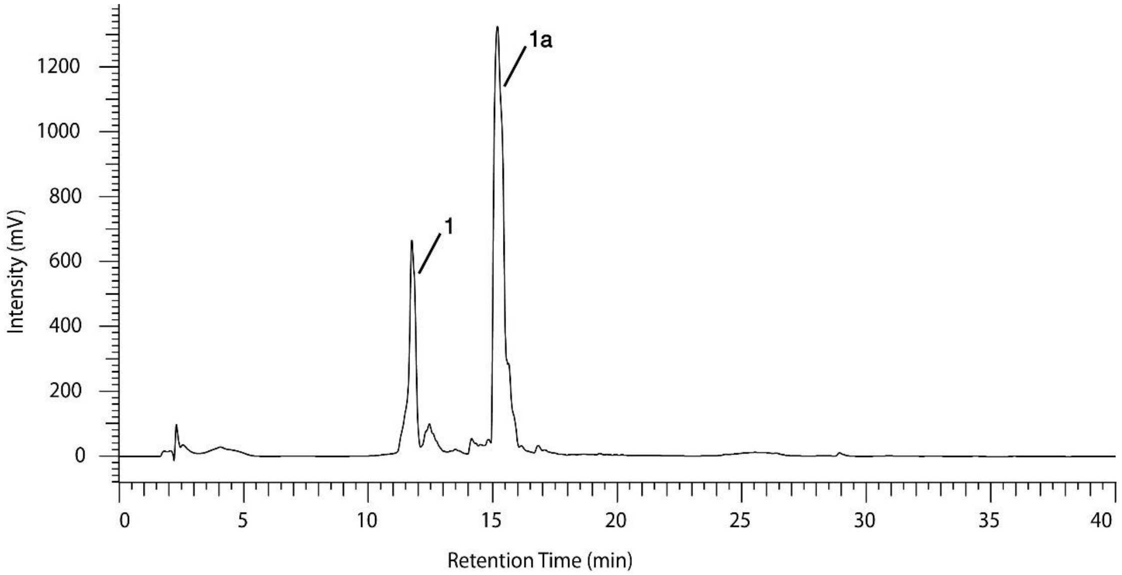

Figure S5. RP-HPLC Chromatogram of crude obtained after sulfonylation of **1** under the conditions described in Table 1 entry 5. (**conditions**: flow rate: 1 mL/min, Vydac analytical RP column (C18, 4.6 × 250 mm, 5 μm), recorded at 220 nm).

Sulfonylation of **1** using conditions described in Table 1 entry 6:

Resin bound motif **1** was sulfonylated to **1a** following the conditions detailed in Table 1 entry 6.

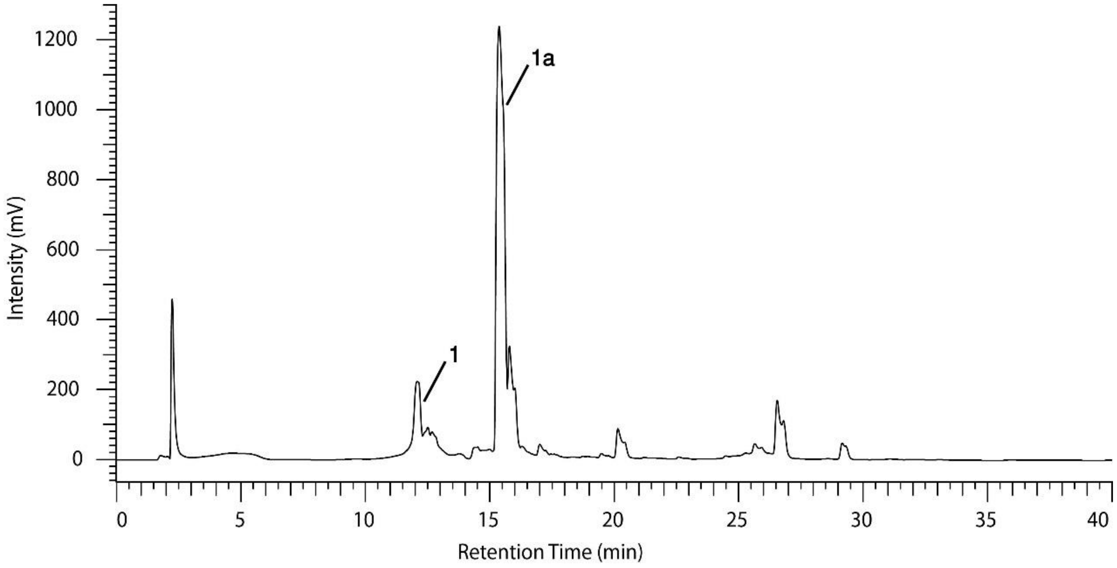

Figure S6. RP-HPLC Chromatogram of crude obtained after sulfonylation of **1** under the conditions described in Table 1 entry 6. (**conditions**: flow rate: 1 mL/min, Vydac analytical RP column (C18, 4.6 × 250 mm, 5 μm), recorded at 220 nm).

Sulfonylation of **1** using conditions described in Table 1 entry 8:

Resin bound motif **1** was sulfonylated to **1a** following the conditions detailed in Table 1 entry 8.

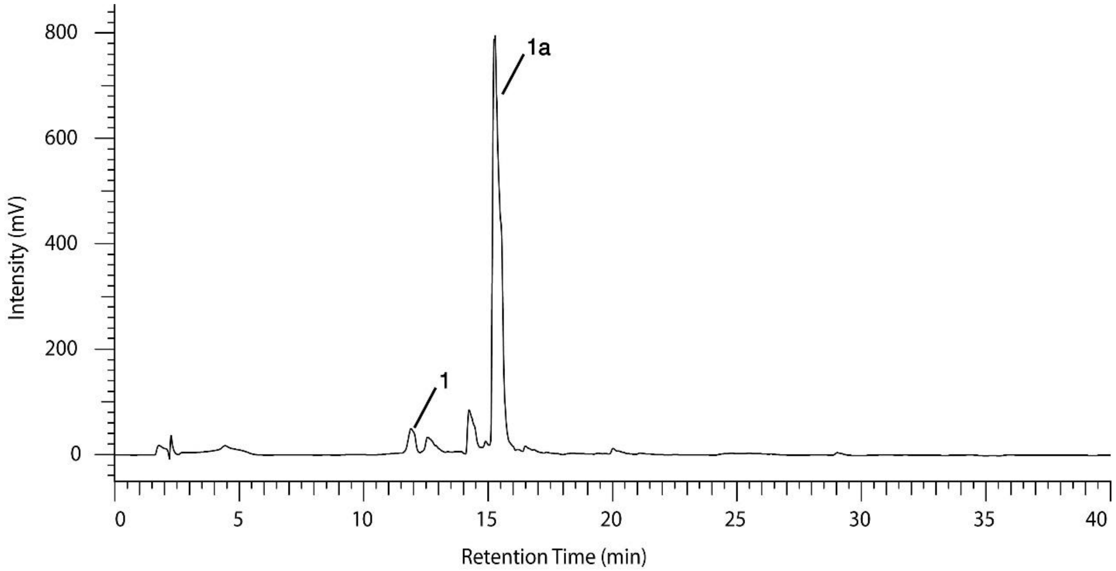

Figure S8. RP-HPLC Chromatogram of crude obtained after sulfonylation of **1** under the conditions described in Table 1 entry 8. (**conditions**: flow rate: 1 mL/min, Vydac analytical RP column (C18, 4.6 × 250 mm, 5 μm), recorded at 220 nm).

2.1.2 Sulfonylation studies of **2** to get **2a**

Sulfonylation of **2** using conditions described in Table 1 entry 9:

Resin bound motif **2** was sulfonylated to **2a** following the conditions detailed in Table 1 entry 9.

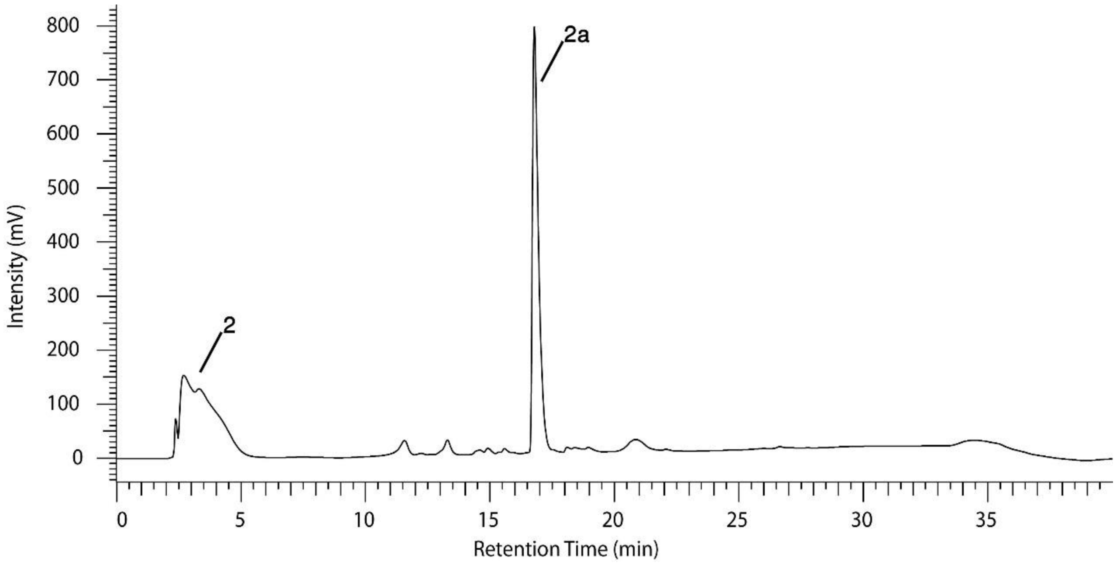

Figure S9. RP-HPLC Chromatogram of crude obtained after sulfonylation of **2** under the conditions described in Table 1 entry 9. (**conditions**: flow rate: 1 mL/min, Vydac analytical RP column (C18, 4.6 × 250 mm, 5 μm), recorded at 220 nm).

<u>Sulfonylation of **2** using conditions described in Table 1 entry 10:</u>

Resin bound motif **2** was sulfonylated to **2a** following the conditions detailed in Table 1 entry 10.

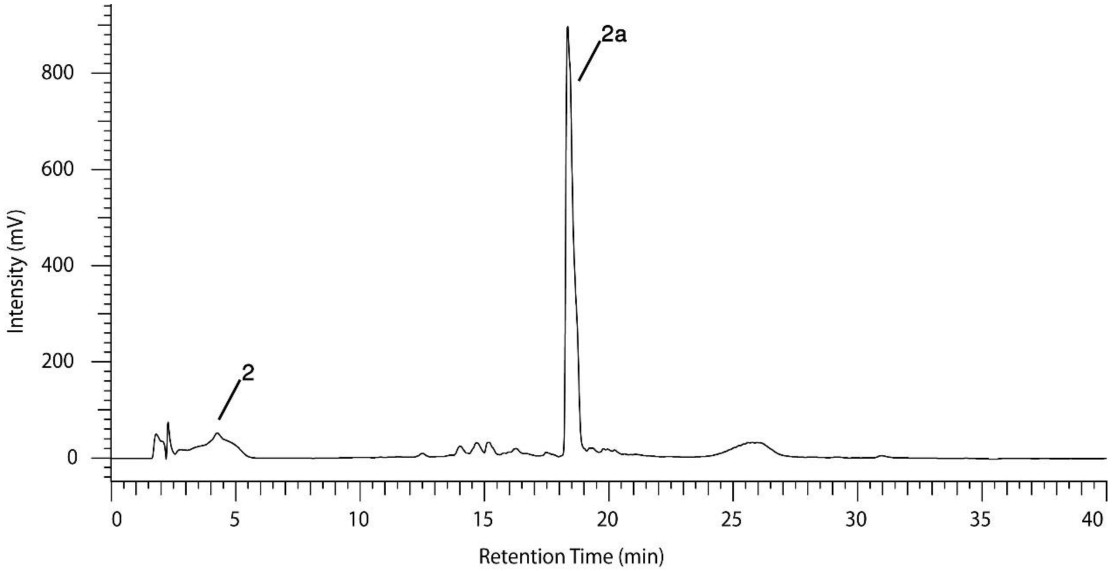

Figure S10. RP-HPLC Chromatogram of crude obtained after sulfonylation of **2** under the conditions described in Table 1 entry 10. (**conditions**: flow rate: 1 mL/min, Vydac analytical RP column (C18, 4.6 × 250 mm, 5 μm), recorded at 220 nm).

2.1.3 Sulfonylation studies of **3** to get **3a**

Sulfonylation of **3** using conditions described in Table 1 entry 11:

Resin bound motif **3** was sulfonylated to **3a** following the conditions detailed in Table 1 entry 11.

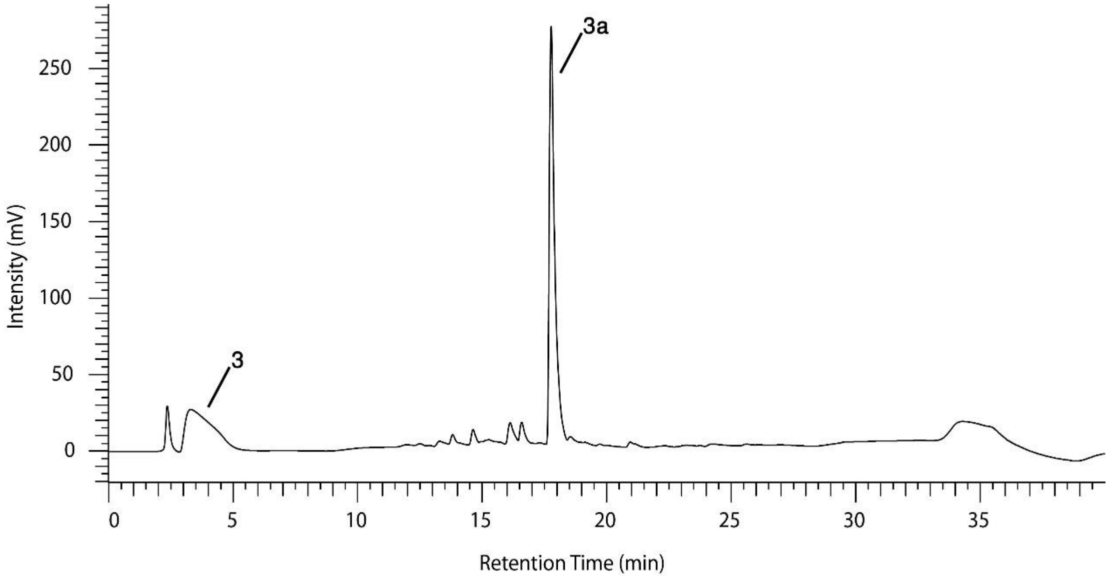

Figure S11. RP-HPLC Chromatogram of crude obtained after sulfonylation of **3** under the conditions described in Table 1 entry 11. (**conditions**: flow rate: 1 mL/min, Vydac analytical RP column (C18, 4.6 × 250 mm, 5 μm), recorded at 220 nm).

Sulfonylation of **3** using conditions described in Table 1 entry 12:

Resin bound motif **3** was sulfonylated to **3a** following the conditions detailed in Table 1 entry 12.

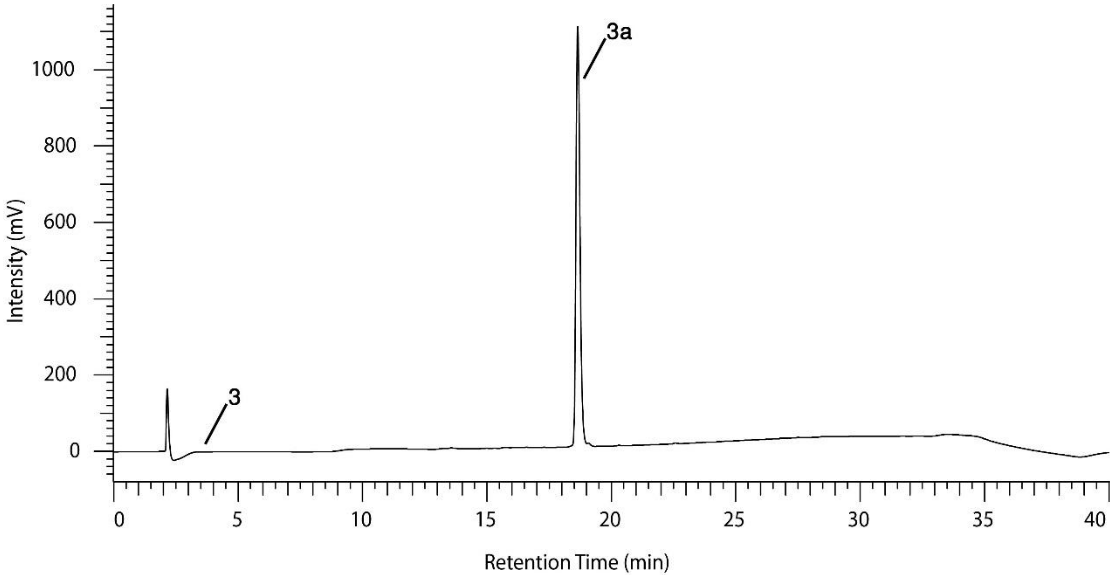

Figure S12. RP-HPLC Chromatogram of crude obtained after sulfonylation of **3** under the conditions described in Table 1 entry 12. (**conditions**: flow rate: 1 mL/min, Vydac analytical RP column (C18, 4.6 × 250 mm, 5 μm), recorded at 220 nm).

2.1.4 Sulfonylation studies of **4** to get **4a**

See Figure 3 in the main article.

2.1.5 Sulfonylation studies of **5** to get **5a**

Sulfonylation of **5** using conditions described in Table 1 entry 17:

Resin bound motif **5** was sulfonylated to **5a** following the conditions detailed in Table 1 entry 17.

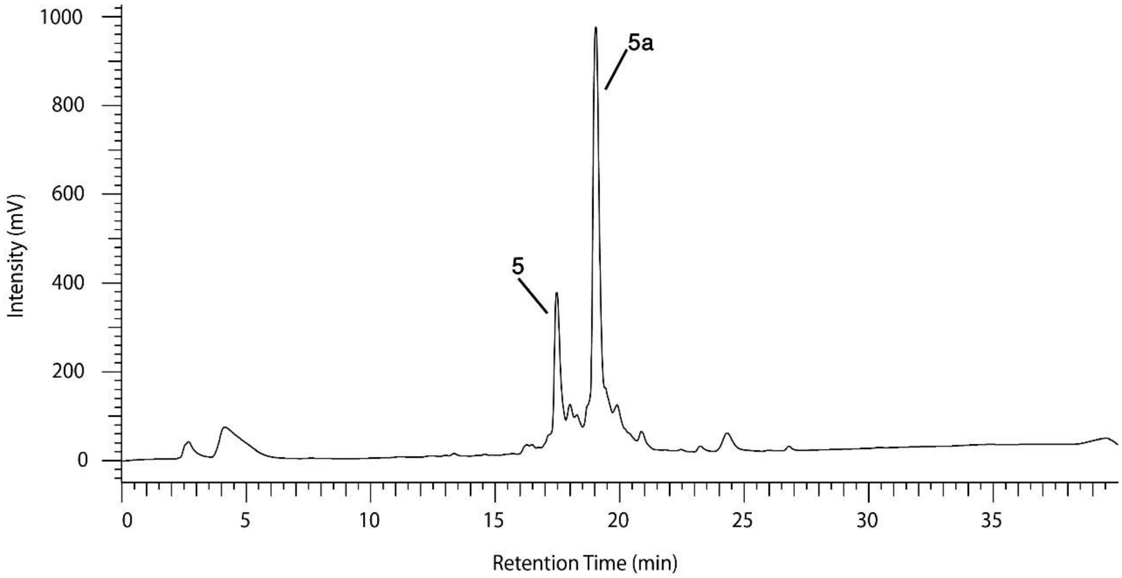

Figure S17. RP-HPLC Chromatogram of crude obtained after sulfonylation of **5** under the conditions described in Table 1 entry 17. (**conditions**: flow rate: 1 mL/min, Vydac analytical RP column (C18, 4.6 × 250 mm, 5 μm), recorded at 220 nm).

Sulfonylation of **5** using conditions described in Table 1 entry 18:

Resin bound motif **5** was sulfonylated to **5a** following the conditions detailed in Table 1 entry 18.

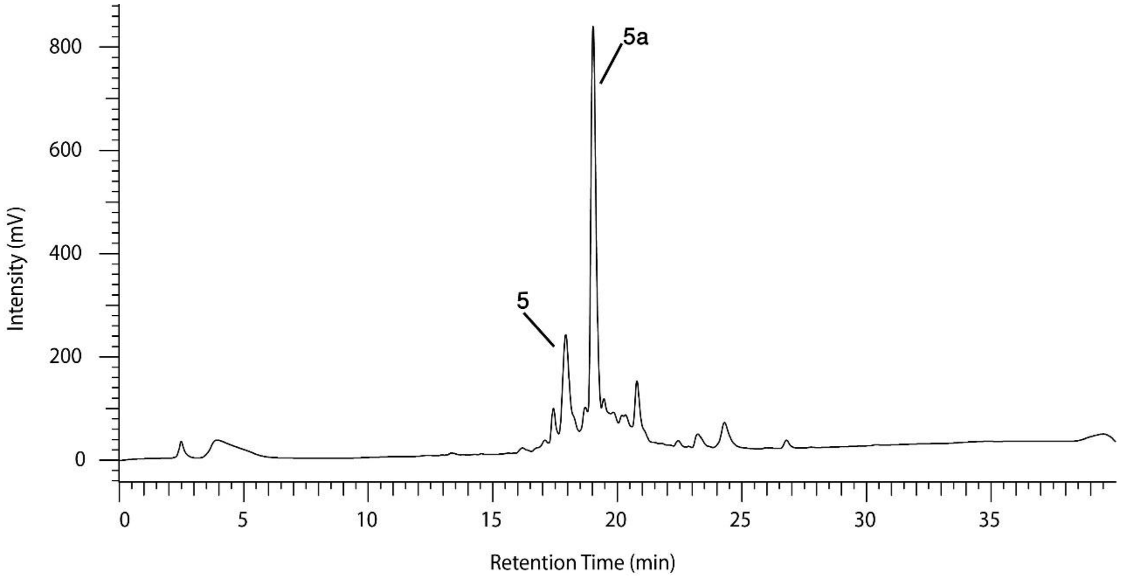

Figure S18. RP-HPLC Chromatogram of crude obtained after sulfonylation of **5** under the conditions described in Table 1 entry 18. (**conditions**: flow rate: 1 mL/min, Vydac analytical RP column (C18, 4.6 × 250 mm, 5 μm), recorded at 220 nm).

2.2 HPLC chromatograms of the crude motifs after the methylation step

In all cases, the crude motifs were removed from solid support after the reaction using either full or small cleavage protocols. The motifs were dissolved in TDW/ACN 1:1 mixture, filtered through a 0.45 μm PTFE filters and injected to an analytical RP-C18-HPLC without any purification.

<u>2.2.1 *N*-methylation of **1a** to get **1b**</u>

Since the methyl group induce only small change in hydrophobicity, only minor difference in the retention time before and after methylation was observed. Nevertheless, the specific peaks that correlate with the products and the reactants were isolated and characterized by mass spectrometry in all cases.

<u>*N*-methylation of **1a** using conditions described in Table 2 entry 1:</u>

Resin bound motif **1a** was *N*-methylated to **1b** following the conditions detailed in Table 2 entry 1.

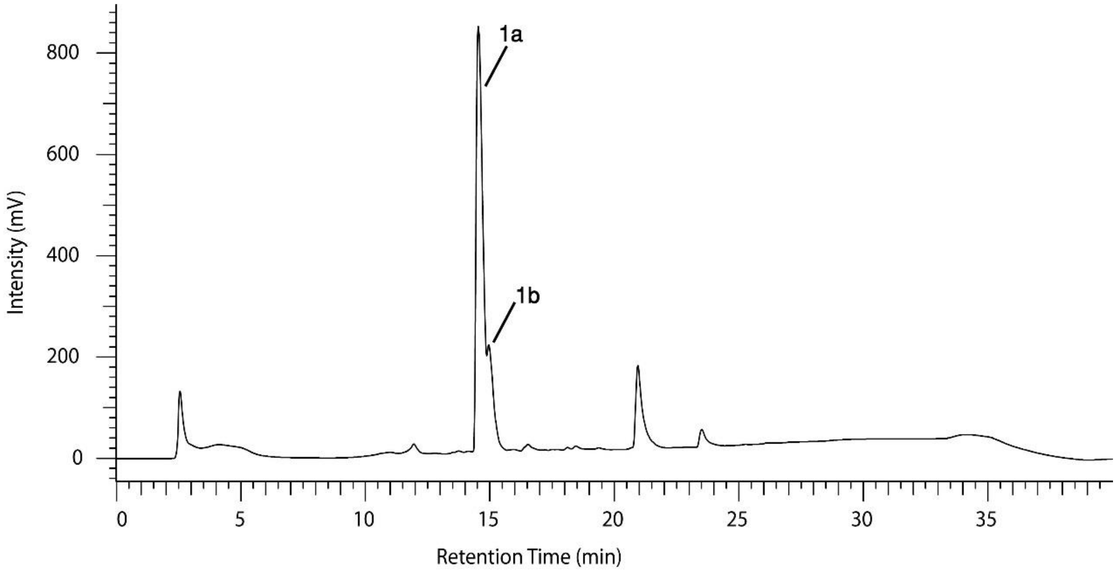

Figure S19. RP-HPLC Chromatogram of crude obtained after *N*-methylation of **1a** under the conditions described in Table 2 entry 1. (**conditions**: flow rate: 1 mL/min, Vydac analytical RP column (C18, 4.6 × 250 mm, 5 μm), recorded at 220 nm).

<u>*N*-methylation of **1a** using conditions described in Table 2 entry 2:</u>

Resin bound motif **1a** was *N*-methylated to **1b** following the conditions detailed in Table 2 entry 2.

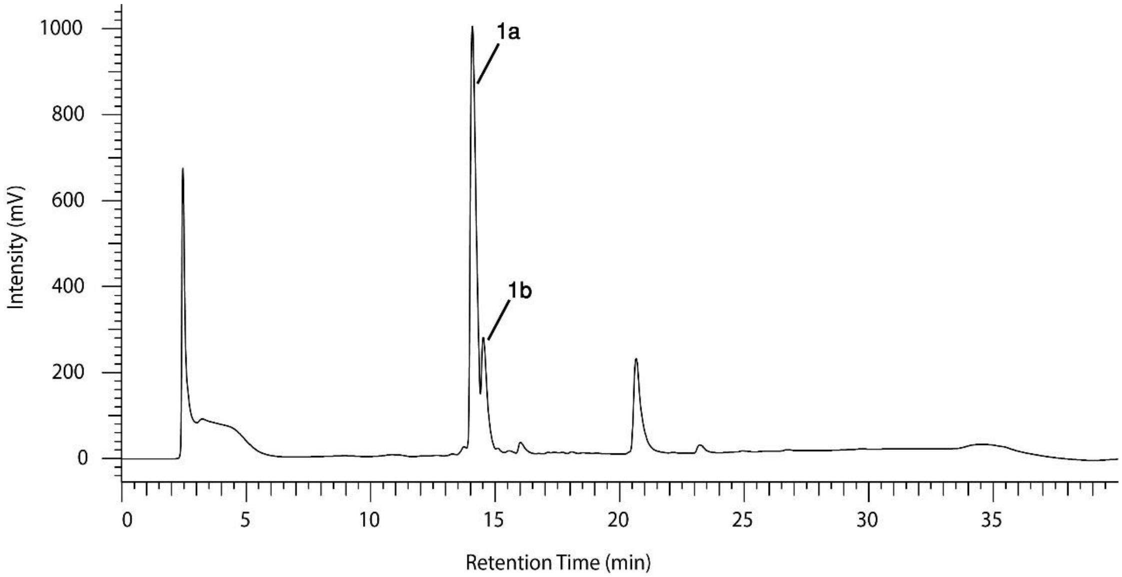

Figure S20. RP-HPLC Chromatogram of crude obtained after *N*-methylation of **1a** under the conditions described in Table 2 entry 2. (**conditions**: flow rate: 1 mL/min, Vydac analytical RP column (C18, 4.6 × 250 mm, 5 μm), recorded at 220 nm).

N-methylation of **1a** using conditions described in Table 2 entry 3:

Resin bound motif **1a** was *N*-methylated to **1b** following the conditions detailed in Table 2 entry 3.

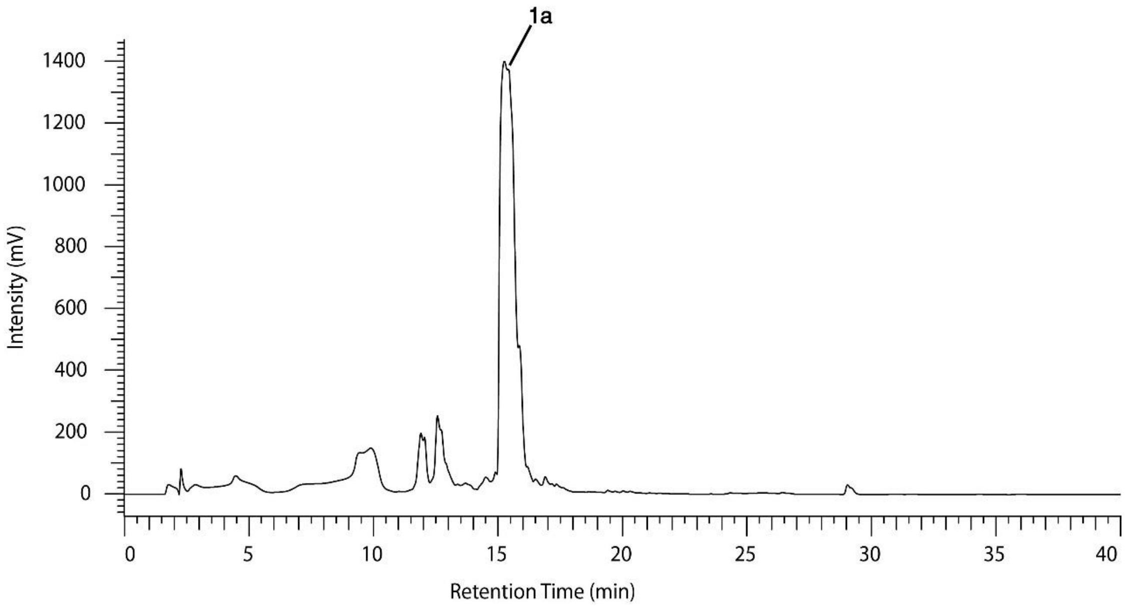

Figure S21. RP-HPLC Chromatogram of crude obtained after *N*-methylation of **1a** under the conditions described in Table 2 entry 3. (**conditions**: flow rate: 1 mL/min, Vydac analytical RP column (C18, 4.6 × 250 mm, 5 μm), recorded at 220 nm).

N-methylation of **1a** using conditions described in Table 2 entry 4:

Resin bound motif **1a** was *N*-methylated to **1b** following the conditions detailed in Table 2 entry 4.

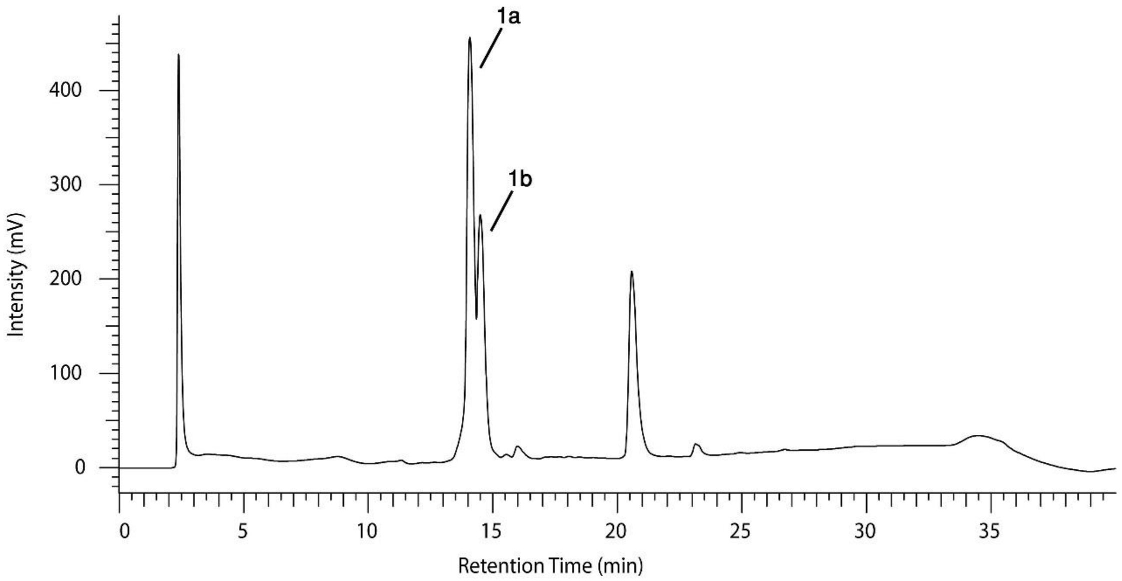

Figure S22. RP-HPLC Chromatogram of crude obtained after *N*-methylation of **1a** under the conditions described in Table 2 entry 4. (**conditions**: flow rate: 1 mL/min, Vydac analytical RP column (C18, 4.6 × 250 mm, 5 μm), recorded at 220 nm).

2.2.2 *N*-methylation of **2a** to get **2b**

N-methylation of **2a** using conditions described in Table 2 entry 5:

Resin bound motif **2a** was *N*-methylated to **2b** following the conditions detailed in Table 2 entry 5.

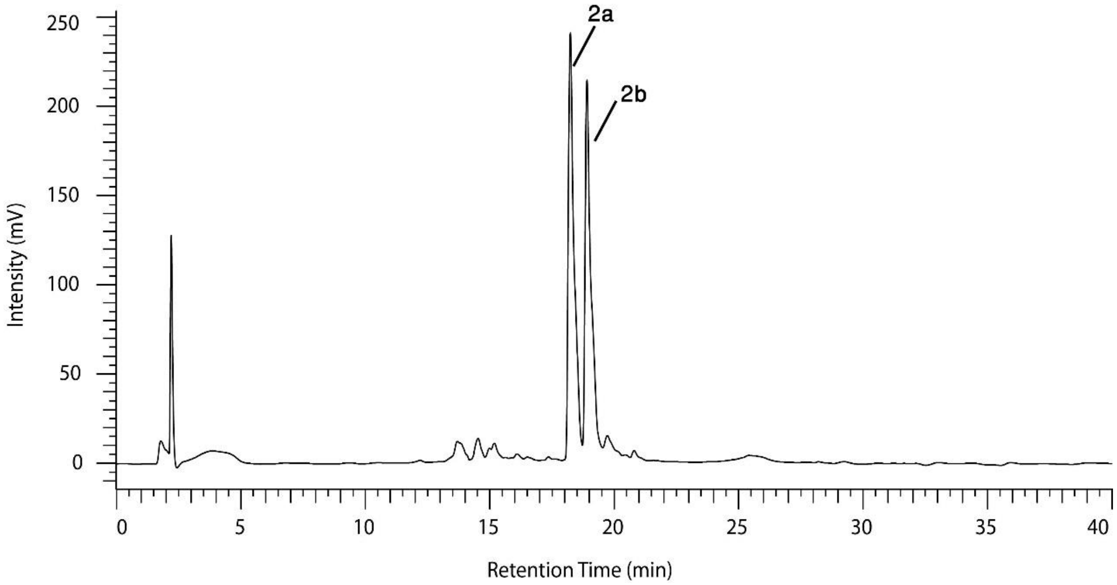

Figure S23. RP-HPLC Chromatogram of crude obtained after *N*-methylation of **2a** under the conditions described in Table 2 entry 5. (**conditions**: flow rate: 1 mL/min, Vydac analytical RP column (C18, 4.6 × 250 mm, 5 μm), recorded at 220 nm).

<u>*N*-methylation of **2a** using conditions described in Table 2 entry 6:</u>

Resin bound motif **2a** was *N*-methylated to **2b** following the conditions detailed in Table 2 entry 6.

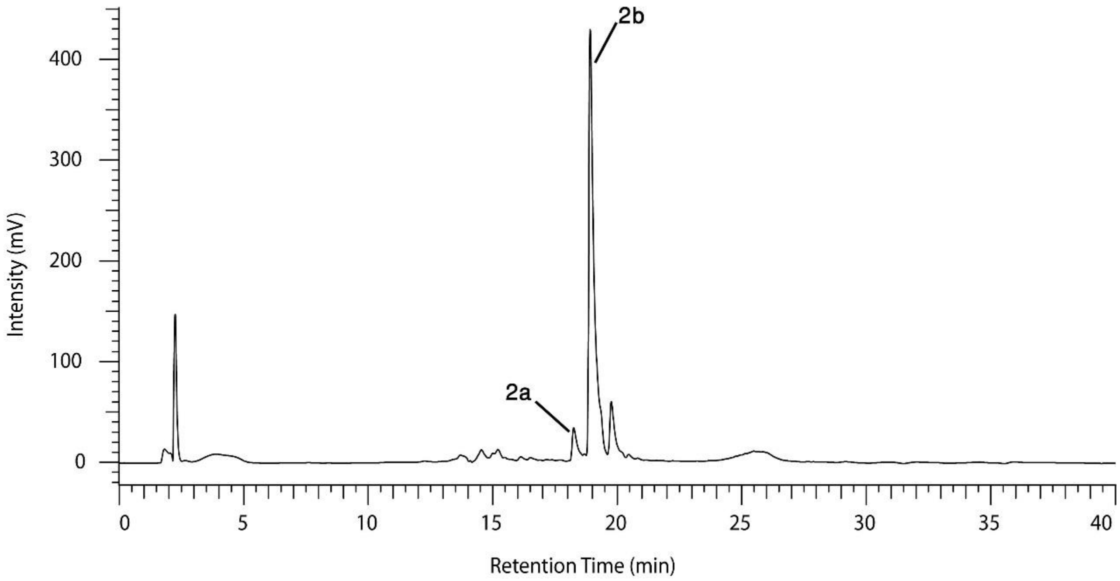

Figure S24. RP-HPLC Chromatogram of crude obtained after *N*-methylation of **2a** under the conditions described in Table 2 entry 6. (**conditions**: flow rate: 1 mL/min, Vydac analytical RP column (C18, 4.6 × 250 mm, 5 μm), recorded at 220 nm).

<u>2.2.3 *N*-methylation of **3a** to get **3b**</u>

See Figure 5 in the main article.

2.2.4 *N*-methylation of **4a** to get **4b**

N-methylation of **4a** using conditions described in Table 2 entry 10:

Resin bound motif **4a** was *N*-methylated to **4b** following the conditions detailed in Table 2 entry 10.

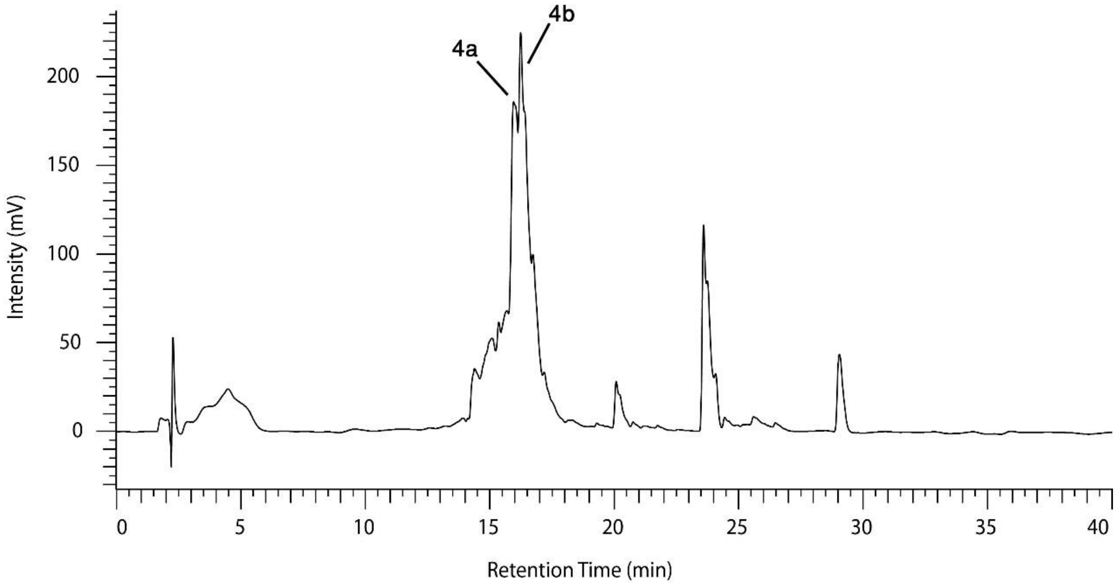

Figure S25. RP-HPLC Chromatogram of crude obtained after *N*-methylation of **4a** under the conditions described in Table 2 entry 10. (**conditions**: flow rate: 1 mL/min, Vydac analytical RP column (C18, 4.6 × 250 mm, 5 μm), recorded at 220 nm).

<u>*N*-methylation of **4a** using conditions described in Table 2 entry 11:</u>

Resin bound motif **4a** was *N*-methylated to **4b** following the conditions detailed in Table 2 entry 11.

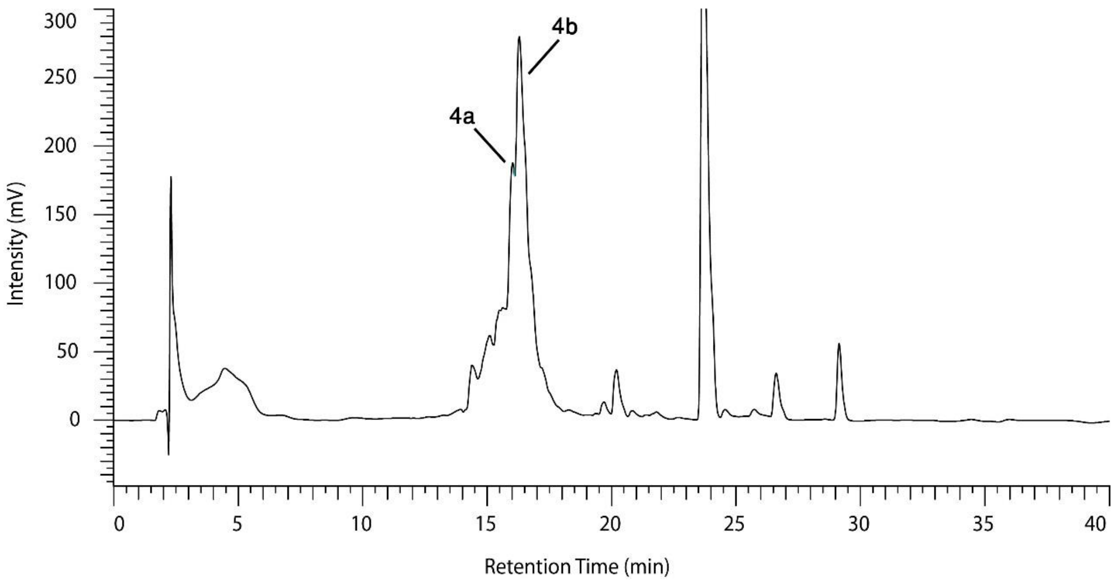

Figure S26. RP-HPLC Chromatogram of crude obtained after *N*-methylation of **4a** under the conditions described in Table 2 entry 11. (**conditions**: flow rate: 1 mL/min, Vydac analytical RP column (C18, 4.6 × 250 mm, 5 μm), recorded at 220 nm).

N-methylation of **4a** using conditions described in Table 2 entry 12:

Resin bound motif **4a** was *N*-methylated to **4b** following the conditions detailed in Table 2 entry 12.

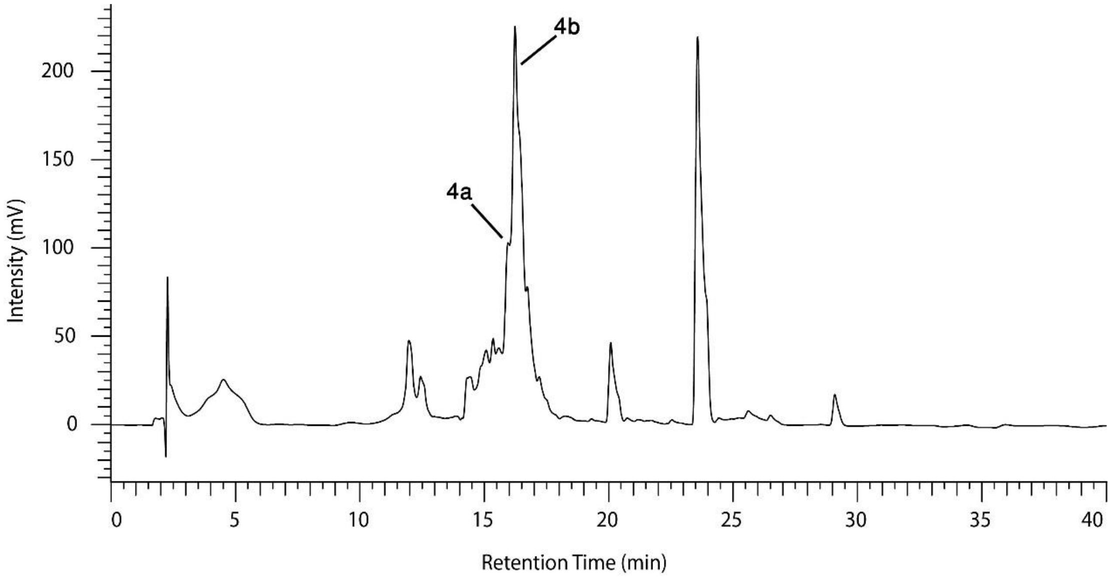

Figure S27. RP-HPLC Chromatogram of crude obtained after *N*-methylation of **4a** under the conditions described in Table 2 entry 12. (**conditions**: flow rate: 1 mL/min, Vydac analytical RP column (C18, 4.6 × 250 mm, 5 μm), recorded at 220 nm).

2.2.5 *N*-methylation of **5a** to get **5b**

N-methylation of **5a** using conditions described in Table 2 entry 13:

Resin bound motif **5a** was *N*-methylated to **5b** following the conditions detailed in Table 2 entry 13.

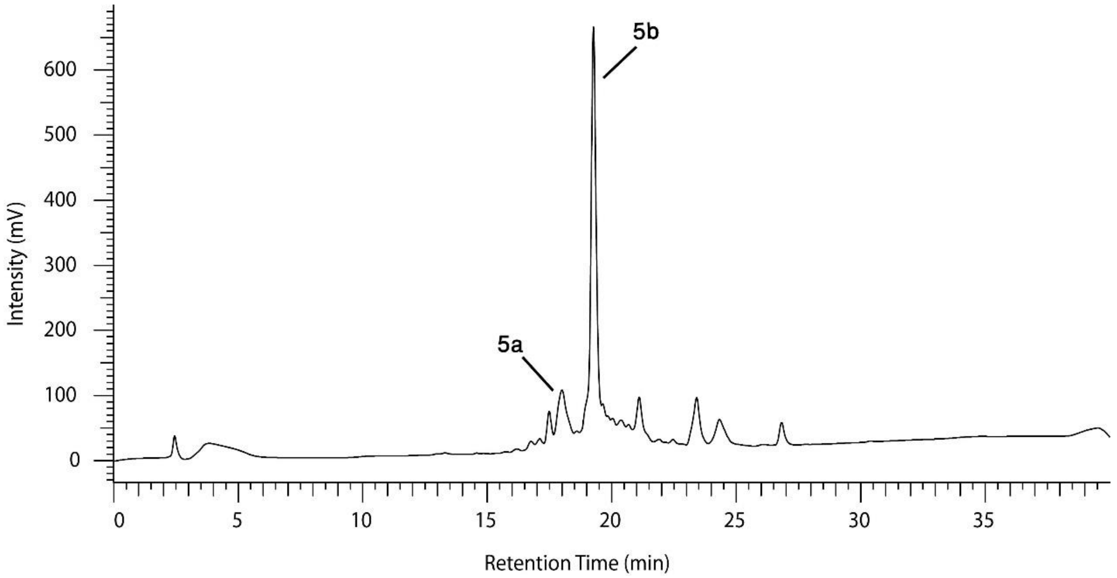

Figure S28. RP-HPLC Chromatogram of crude obtained after *N*-methylation of **5a** under the conditions described in Table 2 entry 13. (**conditions**: flow rate: 1 mL/min, Vydac analytical RP column (C18, 4.6 × 250 mm, 5 μm), recorded at 220 nm).

N-methylation of **5a** using conditions described in Table 2 entry 14:

Resin bound motif **5a** was *N*-methylated to **5b** following the conditions detailed in Table 2 entry 14.

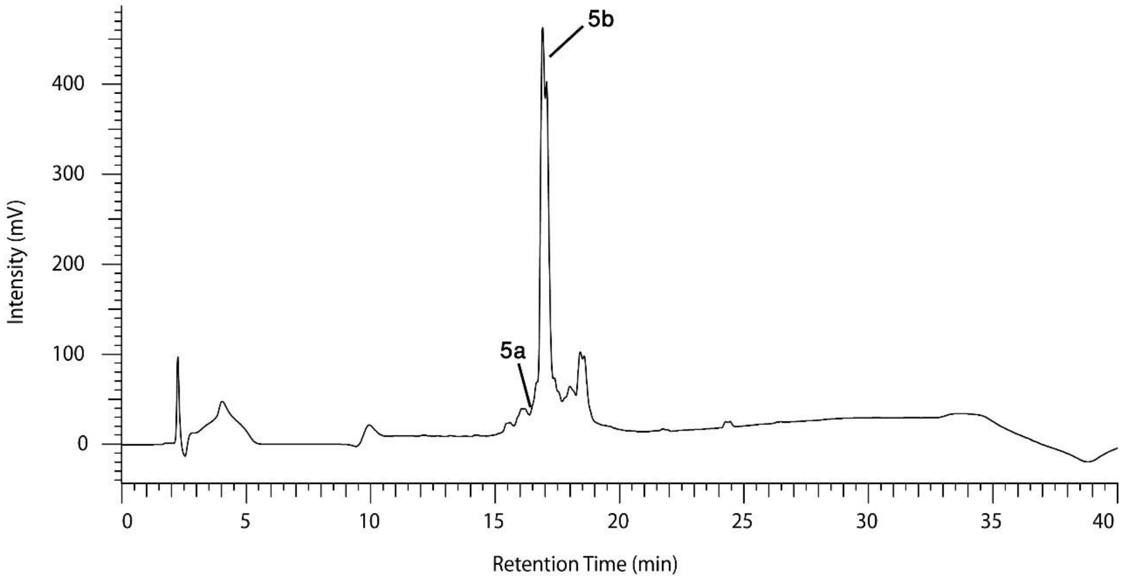

Figure S29. RP-HPLC Chromatogram of crude obtained after *N*-methylation of **5a** under the conditions described in Table 2 entry 14. (**conditions**: flow rate: 1 mL/min, Vydac analytical RP column (C18, 4.6 × 250 mm, 5 μm), recorded at 220 nm).

2.3 *HPLC chromatograms of the crude motifs after the o-NBS removal step*

In all cases, the crude motifs were removed from solid support after the reaction using either full or small cleavage protocols. The motifs were dissolved in TDW/ACN 1:1 mixture, filtered through a 0.45 µm PTFE filters and injected to an analytical RP-C18-HPLC without any purification.

2.3.1 *o*-NBS removal from **2b** to get **2c**

o-NBS removal from **2b** using conditions described in Table 3 entry 1:

o-NBS group was removed from resin bound motif **2b** to get **2c** following the conditions detailed in Table 3 entry 1.

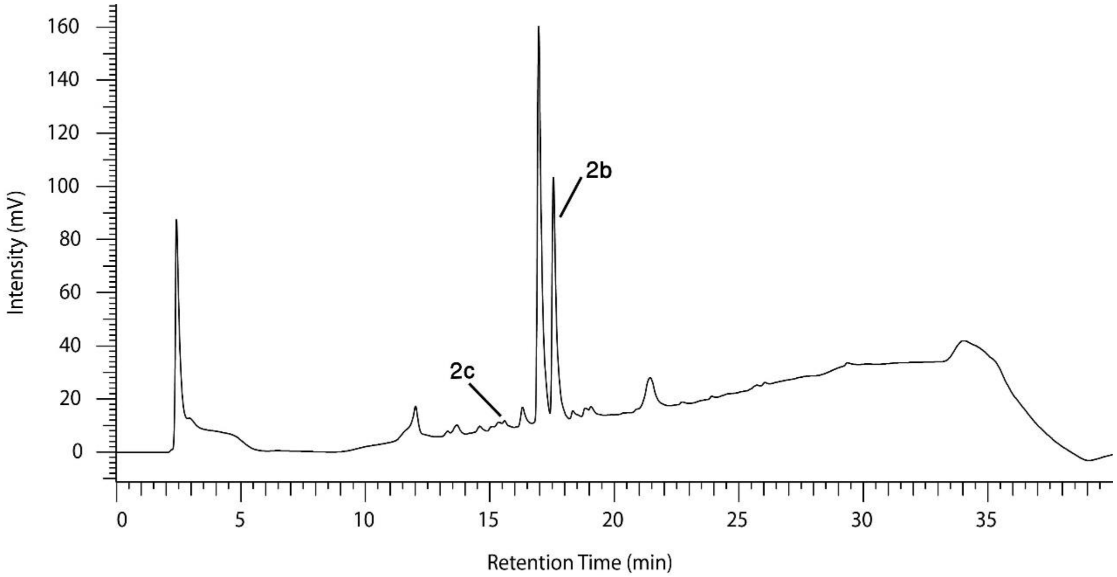

Figure S30. RP-HPLC Chromatogram of crude obtained after *o*-NBS removal from **2b** under the conditions described in Table 3 entry 1. (**conditions**: flow rate: 1 mL/min, Vydac analytical RP column (C18, 4.6 × 250 mm, 5 µm), recorded at 220 nm).

o-NBS removal from **2b** using conditions described in Table 3 entry 2:

o-NBS group was removed from resin bound motif **2b** to get **2c** following the conditions detailed in Table 3 entry 2.

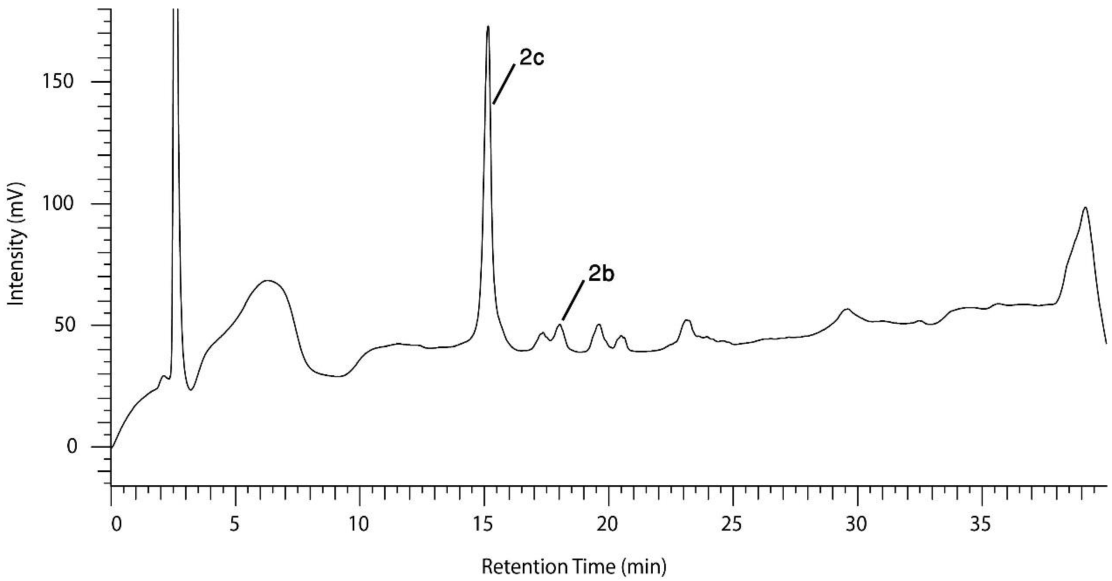

Figure S31. RP-HPLC Chromatogram of crude obtained after *o*-NBS removal from **2b** under the conditions described in Table 3 entry 2. (**conditions**: flow rate: 1 mL/min, Vydac analytical RP column (C18, 4.6 × 250 mm, 5 μm), recorded at 220 nm).

2.3.2 *o*-NBS removal from **3b** to get **3c**

o-NBS removal from **3b** using conditions described in Table 3 entry 3:

o-NBS group was removed from resin bound motif **3b** to get **3c** following the conditions detailed in Table 3 entry 3.

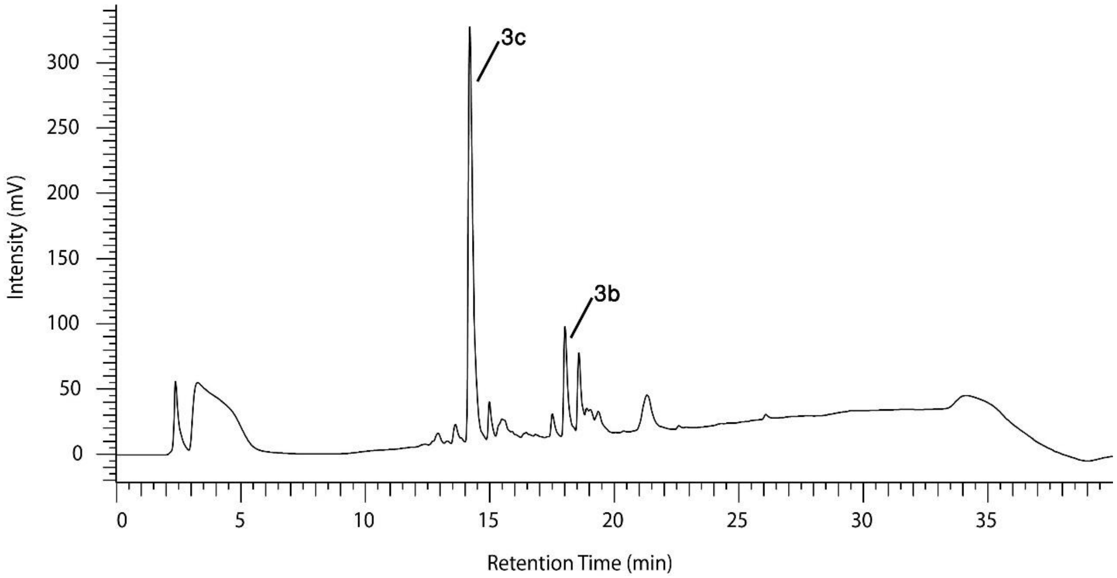

Figure S32. RP-HPLC Chromatogram of crude obtained after *o*-NBS removal from **3b** under the conditions described in Table 3 entry 3. (**conditions**: flow rate: 1 mL/min, Vydac analytical RP column (C18, 4.6 × 250 mm, 5 μm), recorded at 220 nm).

<u>*o*-NBS removal from **3b** using conditions described in Table 3 entry 4:</u>

o-NBS group was removed from resin bound motif **3b** to get **3c** following the conditions detailed in Table 3 entry 4.

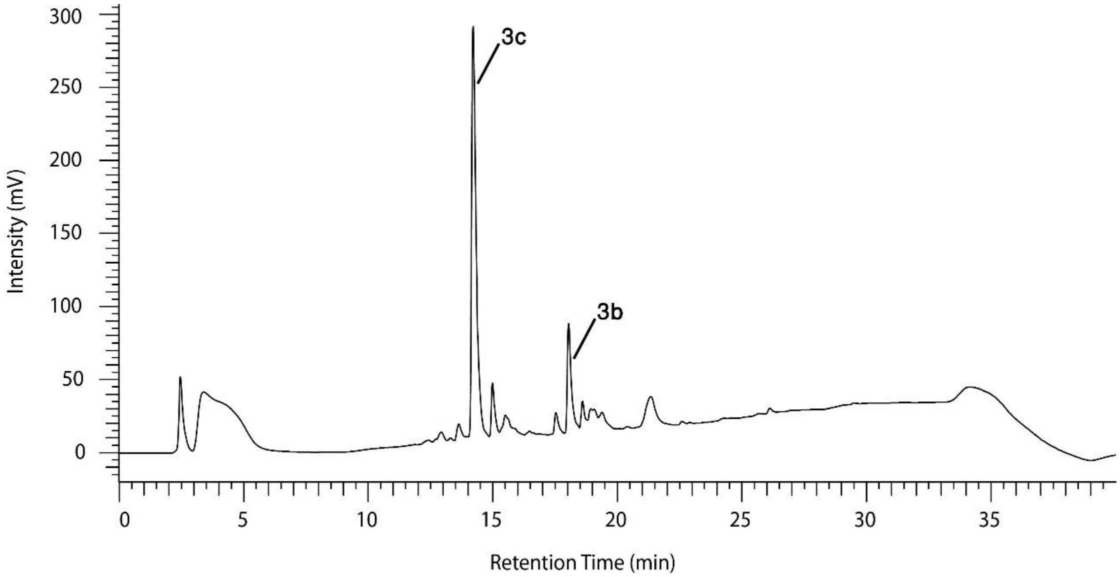

Figure S33. RP-HPLC Chromatogram of crude obtained after *o*-NBS removal from **3b** under the conditions described in Table 3 entry 4. (**conditions**: flow rate: 1 mL/min, Vydac analytical RP column (C18, 4.6 × 250 mm, 5 μm), recorded at 220 nm).

<u>*o*-NBS removal from **3b** using conditions described in Table 3 entry 5:</u>

o-NBS group was removed from resin bound motif **3b** to get **3c** following the conditions detailed in Table 3 entry 5.

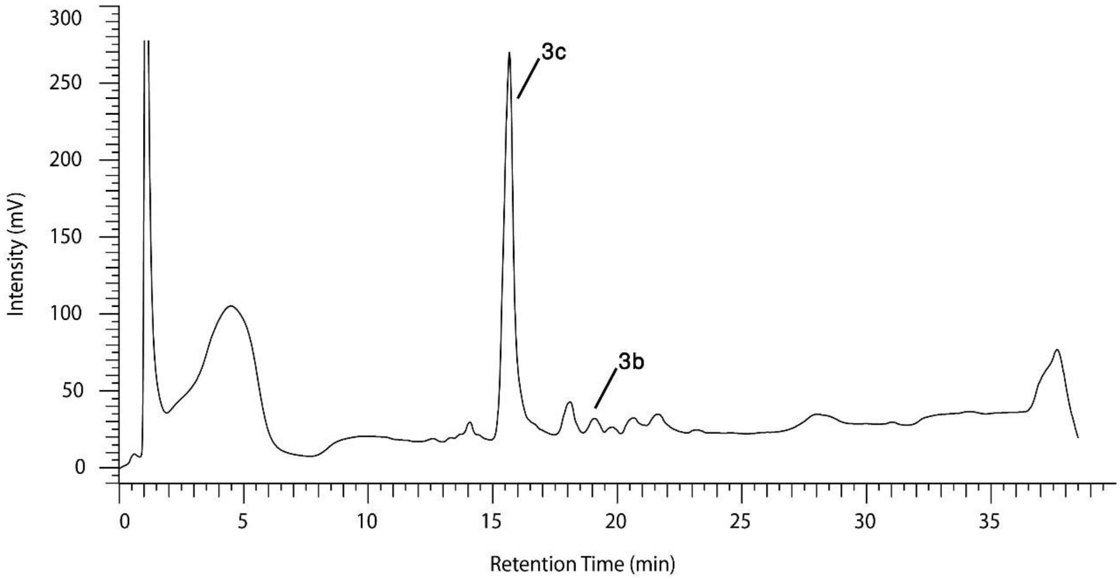

Figure S34. RP-HPLC Chromatogram of crude obtained after *o*-NBS removal from **3b** under the conditions described in Table 3 entry 5. (**conditions**: flow rate: 1 mL/min, Vydac analytical RP column (C18, 4.6 × 250 mm, 5 μm), recorded at 220 nm).

<u>2.3.3 *o*-NBS removal from **5b** to get **5c**</u>

See Figure 6 in the main article.

*2.4 HPLC chromatograms and HRMS characterization of **1SW-1***

*Synthesis of **1SW-1** using Fmoc SPPS*

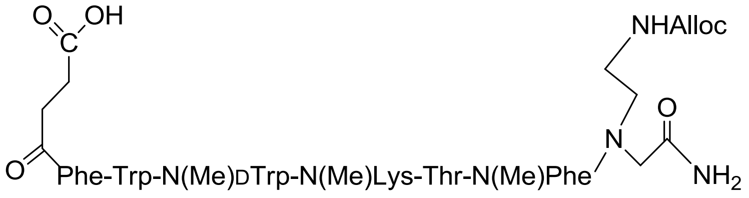

1SW-1

2.4.1 HPLC chromatogram of the crude peptide **1SW-1** synthesized via standard method

1SW-1 was synthesized and N^{α}-methylated at three different sites on resin according to state-of-the-art methods. The peptide was removed from the solid support using TFA cleavage protocol as described above. The crude peptide was centrifuged in diethyl ether, lauphilized overnight and dissolved in TDW/ACN 1:1 mixture, filtered through 0.45 μm PTFE filters and injected to preparative RP-HPLC.

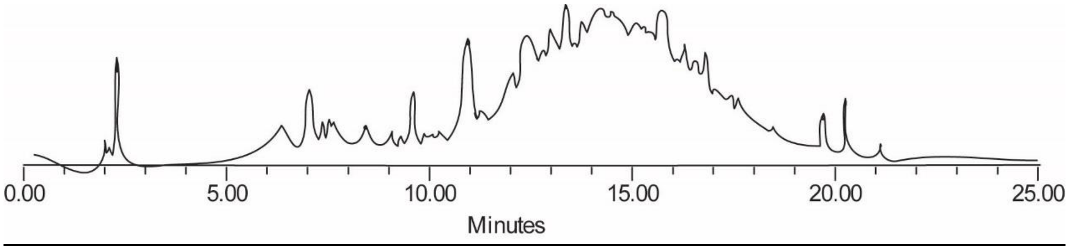

Figure S35. RP-HPLC Chromatogram of crude **1SW-1** which was N^{α}-methylated at three different sites on resin according to state-of-the-art methods. (**conditions**: flow rate: 9 mL/min; Vydac preparative RP column (C18, 22 × 250 mm, 10 μm) recorded at 220 nm).

2.4.2 HPLC chromatogram of the crude peptide **1SW-1** synthesized following the improved new SPS *N*-methylation method

1SW-1 was synthesized and N^{α}-methylated at three different sites on resin according to the new method. The peptide was removed from the solid support using TFA cleavage protocol as described above. The crude peptide was centrifuged in diethyl ether, lauphilized overnight and dissolved in TDW/ACN 1:1 mixture, filtered through 0.45 µm PTFE filters and injected to preparative RP-HPLC.

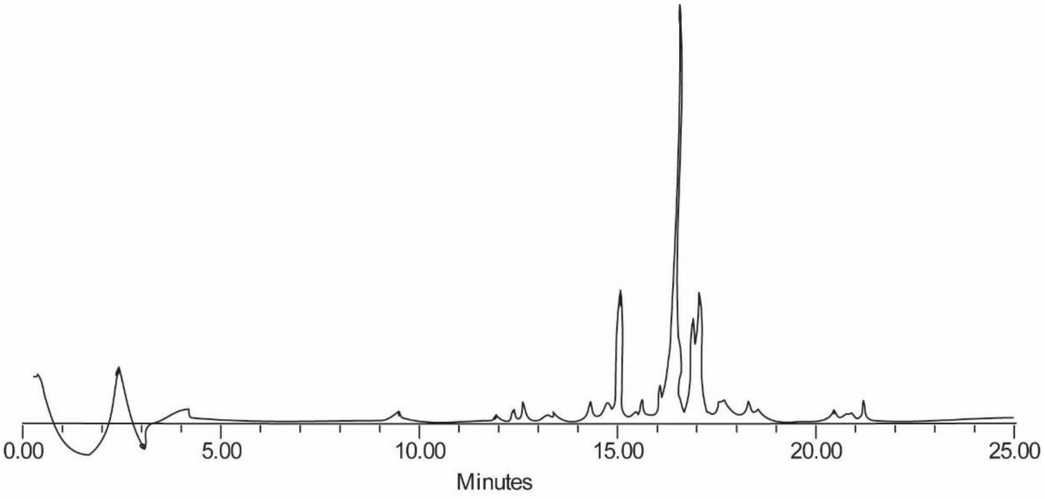

Figure S36. RP-HPLC Chromatogram of crude **1SW-1** which was N^{α}-methylated at three different sites on resin according to the new method**.** (**conditions**: flow rate: 9 mL/min; Vydac preparative RP column (C18, 22 × 250 mm, 10 µm) recorded at 220 nm).

2.4.3 HRMS analysis of isolated **1SW-1** synthesized following the improved new SPS *N*-methylation method

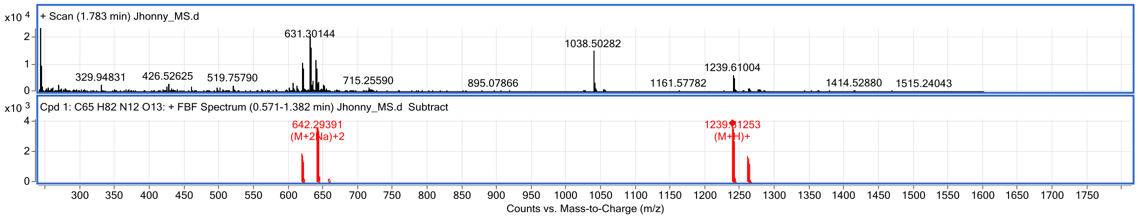

3.0 Tables

3.1 Characterization and detailed reaction conditions of the sulfonylation step

The crude motifs were dissolved in TDW/ACN 1:1 mixture after small cleavage and analyzed by mass spectrometry. The significant peaks in each spectra are presented in Table S1.

Table S1. Characterization of the sulfonylation step

Entry	React.	Prod.	Calc. prod. mass	Obsrv. *m/z*
1	**1**	**1a**	601.21	418.37(r), 587.33(sp), 603.35(p).
2	**1**	**1a**	601.21	416.16(r), 586.94 (sp), 602.98(p).
3	**1**	**1a**	601.21	417.93(r), [498.87, 586.68](sp), 602.75(p).
4	**1**	**1a**	601.21	418.08(r), [499.00, 586.93](sp), 603.17(p).
5	**1**	**1a**	601.21	418.00(r), [498.94, 586.84](sp), 602.83(p).
6	**1**	**1a**	601.21	416.27(r), [499.27, 587.37](sp), 603.47(p).
7	**1**	**1a**	601.21	418.82(r), 790.20(sp).
8	**1**	**1a**	601.21	602.99(p).
9	**2**	**2a**	533.16	[392.19, 431.21](sp), 555.12(p+Na^+).
10	**2**	**2a**	533.16	534.18(p)
11	**3**	**3a**	589.22	487.74(sp), 613.74(p+Na^+), 629.27(p+K^+).
12	**3**	**3a**	589.22	590.25(p)
13	**4**	**4a**	1904.10	1720.05(r), [1776.84, 1800.52, 1891.00](sp), 1905.83(p).
14	**4**	**4a**	1904.10	1904.79(p).
15	**4**	**4a**	1904.10	1720.92(r), 1800.83(sp).
16	**4**	**4a**	1904.10	1720.10(r), [1800.60, 1888.70] (sp), 1905.57(p).
17	**5**	**5a**	1876.20	1693.21(r), 1863.19(sp), 1879.02(p).
18	**5**	**5a**	1876.20	1691.84(r), 1861.87(sp), 1878.21(p).

r = reactant, p = product, sp = side products.

Table S2. Detailed reaction conditions used for the sulfonylation optimization study[a]

Entry	React.	Prod.	*o*-NBS equiv	Base	Base equiv	Time (min)	Cycles	Conv[b].	By-p[c]
1	**1**	**1a**	4	collidine	10	15	1	57%	minor
2	**1**	**1a**	4	collidine	10	15	2	60%	minor
3	**1**	**1a**	4	collidine	10	60	2	73%	major
4	**1**	**1a**	4	collidine	10	120	2	76%	major
5[d,e]	**1**	**1a**	6	collidine	12	15	2	74%	major
6[d]	**1**	**1a**	8	collidine	16	O.N.[f]	1	86%	major
7	**1**	**1a**	4	DBU	10	120	1	N.R.[g]	-
8	**1**	**1a**	4	DMAP	10	120	1	98%	N.D.
9	**2**	**2a**	4	collidine	10	15	2	60%	major
10	**2**	**2a**	4	DMAP	10	120	1	100%	N.D.
11	**3**	**3a**	4	collidine	10	15	2	70%	major
12	**3**	**3a**	4	DMAP	10	120	1	100%	N.D.
13	**4**	**4a**	4	collidine	10	120	1	58%	minor
14	**4**	**4a**	4	DMAP	10	120	1	98%	N.D.
15	**4**	**4a**	4	DBU	10	120	1	N.R.	------
16	**4**	**4a**	4	pyridine	10	120	1	48%	minor
17	**5**	**5a**	4	collidine	10	120	2	74%	minor
18	**5**	**5a**	4	DMAP	10	120	1	89%	N.D.

[a]General optimization conditions: **1–5** (0.35 mmol), *o*-NBS (4 equiv), Base (10 equiv), NMP (5–10 mL), room temperature. [b]conv = conversion to product. Calculated based on: ((area under product peak)/(area under product peak+ area under reactant peak)) × 100%. [c]by-p=by-product: major = significant byproduct; minor = insignificant byproduct; N.D.= no byproduct. [d]higher equivalents of reagents were used. [e]Solvent: DCM. [f]O.N. = overnight.

3.2 Characterization and detailed reaction conditions of the methylation step

The crude motifs were dissolved in TDW/ACN 1:1 mixture after small cleavage and analyzed by mass spectrometry. The significant peaks in each spectra are presented in Table S3.

Table S3. Characterization of the *N*-methylation step

Entry	React.	Prod.	Calc. prod. mass	Obsrv. *m/z*
1	**1a**	**1b**	615.22	[499.13, 587.06](sp), 603.05(r), 617.21(p).
2	**1a**	**1b**	615.22	[249.65, 498.29](sp), 616.24(p).
3	**1a**	**1b**	615.22	587.71(sp), 603.81(r), 617.86(p).
4	**1a**	**1b**	615.22	602.22(r), 616.23(p).
5	**2a**	**2b**	547.17	431.62(sp), 532.54(r) 557.56 (r+Na^+), 571.62(p+Na^+), 587.61(p+K^+).
6	**2a**	**2b**	547.17	431.59(sp), 532.46(r), 571.58(p+Na^+).
7	**3a**	**3b**	603.24	[448.89, 487.98](sp), 628.09(p+Na^+).
8	**3a**	**3b**	603.24	[448.92, 488.15](sp), 628.16(p+Na^+).
9	**3a**	**3b**	603.24	[448.79, 487.95](sp), 628.02(p+Na^+).
10	**4a**	**4b**	1918.13	1891.95(sp), 1907.24(r), 1923.38(p).
11	**4a**	**4b**	1918.13	1892.07(sp), 1906.79(r), 1921.41(p).
12	**4a**	**4b**	1918.13	1889.15(sp), 1904.52(r), 1919.69(p).
13	**5a**	**5b**	1890.17	1877.83(r), 1892.62(p).
14	**5a**	**5b**	1890.17	1876.93(r), 1892.56(p).

r = reactant, p = product, sp = side products.

Table S4. Detailed reaction conditions used for the *N*-methylation step[a]

Entry	React.	Prod.	$(Me)_2SO_4$ equiv	Base	Base equiv	Time[b]	Cycles	Conv.[c]
1	**1a**	**1b**	10	DBU	3	2	2	10%
2	**1a**	**1b**	10	DBU	3	30	1	22%
3[d]	**1a**	**1b**	10	DMAP	3	30	2	N.R.
4	**1a**	**1b**	10	DBU	3	30	2	40%
5	**2a**	**2b**	10	DBU	3	2	2	47%
6	**2a**	**2b**	10	DBU	3	30	2	93%
7	**3a**	**3b**	10	DBU	3	2	2	40%
8	**3a**	**3b**	10	DBU	3	30	1	88%
9	**3a**	**3b**	10	DBU	3	30	2	99%
10	**4a**	**4b**	10	DBU	3	2	2	60%
11	**4a**	**4b**	10	DBU	3	30	1	65%
12	**4a**	**4b**	10	DBU	3	30	2	80%
13	**5a**	**5b**	10	DBU	3	2	2	84%
14	**5a**	**5b**	10	DBU	3	30	2	96%

[a]General optimization conditions: **1a–5a** (0.35 mmol), DBU (3 equiv), NMP (5–10 mL), pre-activation 3 min, addition of $(Me)_2SO_4$ (10 equiv), room temperature. [b]Time, measures in minutes. [c]Conv = conversion to product. Calculated based on: ((area under product peak)/(area under product peak+ area under reactant peak)) × 100%. [d]DMAP was used as base.

3.3 Characterization and detailed reaction conditions of the o-NBS removal step

The crude motifs were dissolved in TDW/ACN 1:1 mixture after small cleavage and analyzed by mass spectrometry. The significant peaks in each spectra are presented in Table S5.

Table S5. Characterization of the *o*-NBS removal step

Entry	React.	Prod.	Calc. prod. mass	Observed *m/z*
1	**2b**	**2c**	362.20	363.85(p), [391.83, 430.86](sp).
2	**2b**	**2c**	362.20	363.21(p)
3	**3b**	**3c**	418.26	420.40(p), 448.52(sp).
4	**3b**	**3c**	418.26	420.58(p), [448.63, 487.68](sp).
5	**3b**	**3c**	418.26	419.26(p)
6	**5b**	**5c**	1705.17	1705.39(p), 1876.82(sp), 1891.56(r).
7	**5b**	**5c**	1705.17	1707.4(p).

r = reactant, p = product, sp = side products.

Table S6. Detailed reaction conditions used for the *o*-NBS removal step[a]

Entry	React.	Prod.	$HO(CH_2)_2SH$	Base	Base equiv	Time[b]	Cycles	Conv.[c]
1	**2b**	**2c**	10	DBU	5	5	2	10%
2	**2b**	**2c**	10	DBU	5	30	2	98%
3	**3b**	**3c**	10	DBU	5	5	2	80%
4[d]	**3b**	**3c**	14	DBU	7	5	2	80%
5	**3b**	**3c**	10	DBU	5	30	1	98%
6	**5b**	**5c**	10	DBU	5	5	2	16%
7	**5b**	**5c**	10	DBU	5	30	2	100%

[a]General optimization conditions: **2b, 3b, 5b** (0.35 mmol), DBU (5 equiv), 2-mercaptoethanol (10 equiv), NMP (5–10 mL), room temperature. [b]Time, measures in minutes. [c]Conv = conversion to product, calculated based on: ((area under product peak)/(area under product peak+ area under reactant peak)) × 100%. [d]Higher equivalents of reagents were used.

References

1. Chandra, K.; Roy, T. K.; Naoum, J. N.; Gilon, C.; Gerber, R. B.; Friedler, A., A highly efficient in situ N-acetylation approach for solid phase synthesis. *Org. Biomol. Chem.* **2014,** *12* (12), 1879-1884.
2. Hurevich, M.; Tal-Gan, Y.; Klein, S.; Barda, Y.; Levitzki, A.; Gilon, C., Novel method for the synthesis of urea backbone cyclic peptides using new Alloc-protected glycine building units. *J. Pept. Sci.* **2010,** *16* (4), 178-185.
3. Kaiser, E.; Colescot.Rl; Bossinge.Cd; Cook, P. I., Color Test for Detection of Free Terminal Amino Groups in Solid-Phase Synthesis of Peptides. *Anal. Biochem.* **1970,** *34* (2), 595-&.
4. Kay, C.; Lorthioir, O. E.; Parr, N. J.; Congreve, M.; McKeown, S. C.; Scicinski, J. J.; Ley, S. V., Solid-phase reaction monitoring - Chemical derivatization and off-bead analysis. *Biotechnol. Bioeng.* **2000,** *71* (2), 110-118.
5. Vojkovsky, T., Detection of Secondary-Amines on Solid-Phase. *Peptide Res.* **1995,** *8* (4), 236-237.
6. Ahlrichs, R.; Bar, M.; Haser, M.; Horn, H.; Kolmel, C., Electronic-Structure Calculations on Workstation Computers - the Program System Turbomole. *Chem. Phys. Lett.* **1989,** *162* (3), 165-169.
7. Dunning, T. H., Gaussian-Basis Sets for Use in Correlated Molecular Calculations .1. The Atoms Boron through Neon and Hydrogen. *J. Chem. Phys.* **1989,** *90* (2), 1007-1023.
8. Parr, R. G.; Weitao, Y., *Density-functional theory of atoms and molecules*. Oxford University Press: New York, 1989.
9. Stephens, P. J.; Devlin, F. J.; Chabalowski, C. F.; Frisch, M. J., Ab-Initio Calculation of Vibrational Absorption and Circular-Dichroism Spectra Using Density-Functional Force-Fields. *J. Phys. Chem.* **1994,** *98* (45), 11623-11627.
